# The Electrical Aftermath: Brain Signals of Posttraumatic Stress Disorder Filtered Through a Clinical Lens

**DOI:** 10.3389/fpsyt.2019.00368

**Published:** 2019-05-31

**Authors:** Mamona Butt, Elizabeth Espinal, Robin L. Aupperle, Valentina Nikulina, Jennifer L. Stewart

**Affiliations:** ^1^Department of Psychology, Queens College, City University of New York, Flushing, NY, United States; ^2^Laureate Institute for Brain Research, Tulsa, OK, United States; ^3^Department of Community Medicine, Oxley College of Health Sciences, University of Tulsa, Tulsa, OK, United States; ^4^Department of Psychology, The Graduate Center, City University of New York, New York, NY, United States

**Keywords:** posttraumatic stress disorder, trauma, electroencephalography, event related potentials, brain asymmetry

## Abstract

This review aims to identify patterns of electrical signals identified using electroencephalography (EEG) linked to posttraumatic stress disorder (PTSD) diagnosis and symptom dimensions. We filter EEG findings through a clinical lens, evaluating nuances in findings according to study criteria and participant characteristics. Within the EEG frequency domain, greater right than left parietal asymmetry in alpha band power is the most promising marker of PTSD symptoms and is linked to exaggerated physiological arousal that may impair filtering of environmental distractors. The most consistent findings within the EEG time domain focused on event related potentials (ERPs) include: 1) exaggerated frontocentral responses (contingent negative variation, mismatch negativity, and P3a amplitudes) to task-irrelevant distractors, and 2) attenuated parietal responses (P3b amplitudes) to task-relevant target stimuli. These findings suggest that some individuals with PTSD suffer from attention dysregulation, which could contribute to problems concentrating on daily tasks and goals in lieu of threatening distractors. Future research investigating the utility of alpha asymmetry and frontoparietal ERPs as diagnostic and predictive biomarkers or intervention targets are recommended.

## Posttraumatic Stress and the Brain: The Electrical Aftermath

Approximately 6 out of 100 people in the U.S. will suffer from posttraumatic stress disorder (PTSD) during their lifetime, an illness linked to significant distress, disability, and social/emotional impairment (1). Although theoretical and data-driven approaches suggest that PTSD is linked to alterations in brain circuitry that involve subcortical reactivity to trauma-related memories, thoughts, and emotions in addition to impaired prefrontal emotion regulation and inhibitory control (2–5), there are still gaps in our knowledge that neuroimaging tools can be used to address. This includes: 1) What is the extent of perceptual, cognitive, and emotional alterations as a function of trauma symptoms with respect to processing delays and allocation of brain resources? 2) To what extent do these brain alterations predict future course of disorder as well as treatment success or failure? and 3) Can these particular alterations be targeted by psychological and/or biological treatment approaches? A goal of clinical neuroscience is to develop precision medicine for individualized monitoring and treatment of clinical symptoms (6). With respect to treatment dissemination, it would be ideal if symptom and treatment evaluation tools were inexpensive and widely available. In this respect, electroencephalography (EEG) is an ideal methodology for moving the field towards a neuroscience-based, precision medicine approach to the treatment of PTSD.

Clinical neuroscientists often employ EEG, a relatively inexpensive yet powerful neuroimaging tool, to measure electrical brain signals in hopes of uncovering mechanisms and circuitry that are disrupted as a function of PTSD that can in turn be utilized in prevention, screening, and intervention efforts. Employing EEG methodology in clinical populations is beneficial for several reasons. First, EEG is a non-invasive, easy to administer technique that measures electrical signals on the scalp arising from pyramidal neurons firing within the cortex. Second, EEG possesses excellent temporal resolution on the order of milliseconds, facilitating study of early perceptual, attentional, and cognitive/emotional processes that may be derailed as a function of disorder. Third, comprehensive large-scale EEG electrode configurations are now available, providing improved spatial resolution to localize these electrical signals within the brain, elucidating where and when mental processes occur and how they are disrupted in particular clinical populations. Fourth, as mobile EEG systems are also available, recording of electrical signals can be routinely employed in clinical inpatient/outpatient, hospital, and community settings to assess changes as a function of symptom severity or improvement.

The purpose of this review is to evaluate what EEG research has taught us thus far with respect to brain circuitry and processes disrupted as a function of traumatic events and PTSD, results that we term “the electrical aftermath” of trauma, and then suggest future directions to further explore the unanswered questions presented above. To this end, we first define trauma and PTSD, explain how these constructs are typically measured within the EEG literature, and present clinical issues relevant to EEG studies of PTSD. Second, we evaluate studies of trauma and PTSD in both frequency and time domains of EEG recording, highlighting consistent findings while casting a floodlight on clinical and methodological conundrums warranting further consideration in future studies. Lastly, we discuss research evaluating EEG signals as potential treatment-relevant biomarkers, and the research that is necessary for moving towards the use of EEG to enhance treatment outcomes for PTSD.

## Defining Trauma and PTSD

As the majority of the studies discussed within this review define PTSD on the basis of *Diagnostic and Statistical Manual of Mental Disorders—IV* (DSM-IV) criteria, we will first outline these criteria and then elaborate upon recent DSM-5 revisions. PTSD is the primary disorder characterized by exposure to a traumatic event, wherein one encounters or witnesses actual or threatened death or severe injury and reacts with intense fear, helplessness, and/or horror (7). Traumatic events include child abuse (physical, sexual, emotional), child neglect, domestic violence, physical/sexual assault, accidents, life-threatening illness, death of a family member, natural disasters, war, combat, and community or school violence (8). Other DSM-IV PTSD criteria include at least one symptom of recurrent and intrusive thoughts (e.g., flashbacks), three or more symptoms of persistent avoidance and numbing of stimuli related to the trauma (e.g., avoiding thoughts and activities), and two or more symptoms of increased arousal (e.g., hypervigilance and difficulty falling/staying asleep) for at least 1 month. Symptoms of re-experiencing include recurrent nightmares, physical responses to trauma cues, and/or sensations that the trauma is continuing. For example, Iraqi veterans may have frequent nightmares of their experiences in Iraq, sweating every time they remember particular situations such as bombings; additionally, veterans may avoid visiting places similar to Iraq to avoid aversive feelings, memories, and physiological sensations linked to these violent attacks. A PTSD diagnosis requires that symptoms cause significant disturbance and impairment to social, vocational, and other imperative life functioning (7). PTSD criteria in DSM-5 (9) were updated by transforming these three clusters (re-experiencing, avoidance/numbing, and arousal) into four symptom clusters present for 1 month or longer: 1) intrusions; 2) avoidance of trauma-related thoughts or activities; 3) negative alterations in cognition and mood (e.g., negative affect, anhedonia, self- and other-blame, inability to recall details of the trauma, and isolation); and 4) changes in arousal (e.g., hypervigilance, exaggerated startle response, irritability, aggression, sleep problems, and concentration difficulties). One important issue to consider with respect to categorical classification of disorder is that there are many paths to a PTSD diagnosis, and symptom heterogeneity is the rule, not the exception (10, 11). As a result, in addition to evaluating brain activity as a function of presence versus absence of a PTSD diagnosis, relating brain processes to dimensional symptom presentations in line with the Research Domain Criteria may facilitate more rapid development of precision medicine for treatment of trauma-related dysfunction (12–14).

## Clinically Relevant Issues to Explore

A primary aim of clinical neuroscience research is to identify brain circuitry and/or processes (often termed “biological markers” or “biomarkers”) that differentiate individuals with and without a particular illness or condition of interest. Three biomarker types are especially relevant to trauma neuroscience: 1) diagnostic, identifying people with a specific disorder diagnosis (or subtype of disorder); 2) predictive, identifying people who will improve or worsen as a result of treatment; and 3) prognostic, identifying people who relapse or experience changes in clinical severity (15). Traditionally, researchers advocated for diagnostic metrics of disorder that were specific (i.e., differentiating those with PTSD from healthy individuals without these symptoms) and sensitive (i.e., discerning those with PTSD from individuals diagnosed with other disorders); biomarkers could also be conceptualized as state-like (evident only in people meeting criteria for current PTSD) or trait-like (present in people who have ever suffered from PTSD, regardless of current symptom status). However, in line with the dimensional Research Domain Criteria (RDoC) framework, biomarkers may index clinical symptoms that transcend diagnostic boundaries (e.g., concentration difficulties, negative affect, and sleep disturbances) and that may predict illness course (prognostic biomarkers) and treatment outcome (predictive biomarkers). Biomarker strength may also vary as a function of biological differences such as sex or age. Comparing patterns of brain activity between individuals diagnosed with PTSD and healthy individuals who have experienced similar traumatic events may help to identify what brain mechanisms capture clinically heightened responses to trauma (not just the experience of trauma itself). Moreover, it is useful to identify whether patterns of electrical signals are a result of a particular type of trauma (e.g., combat) or are present across various types of trauma (e.g., sexual assault, childhood neglect, car accidents, natural disasters).

It is unlikely that a diagnostic biomarker of “pure PTSD” exists, given that PTSD symptoms are also present in major depressive disorder (MDD) (negative, affect, anhedonia, sleep/concentration problems), panic disorder (PD) (negative affect, hyperarousal), and generalized anxiety disorder (GAD) (negative affect, irritability, sleep/concentration difficulties); in addition, avoidance and negative affect associated with reactions to trauma in PTSD are often accompanied by coping strategies involving substance use, thereby complicating the clinical picture (16, 17). Individuals suffering from PTSD have approximately twice the odds of meeting criteria for comorbid PD, GAD, and MDD than those without the disorder (1). Prior work indicates that although PTSD and MDD share symptoms of distress, they are distinct disorders, and concurrent experience of both is associated with greater re-experiencing and negative affect than PTSD alone (18). Given symptom overlap and high comorbidity, it is essential for clinical neuroscience work to explicitly address potentially comorbid symptoms and disorders to address biomarkers of transdiagnostic symptoms (e.g., anhedonia, anxiety) as opposed to just PTSD-specific symptoms (e.g., intrusions involving nightmares and flashbacks). The majority of EEG studies discussed below investigate potential diagnostic biomarkers of PTSD and trauma symptoms; less electrophysiology work has focused on proposed prognostic or predictive biomarkers within the context of trauma treatment or symptom course over time.

Similarly, with regard to state versus trait prognostic and diagnostic biomarkers, it is helpful for neuroscience research to determine whether current/past symptom status, current medication/treatment status, and time since the traumatic event moderates relationships between brain mechanisms and PTSD. Finally, concerning potential sex differences, PTSD is more prevalent in women than men (1) and it is possible that differences in stress-related biological processes may clarify sex imbalances in PTSD prevalence (19). Given these concerns, the present review identifies the degree to which EEG studies of PTSD and trauma more broadly address issues of comorbidity, medication status, time elapsed since the traumatic event, and sex differences within clinical groups as well as degree of trauma experienced in healthy comparison subjects. Evaluation of these factors enable us to provide recommendations for future PTSD biomarker refinement and testing. Before relating clinical symptoms to electrophysiology, however, we first explain how trauma and PTSD are quantified within this literature.

## Clinical Capture of Trauma and Posttraumatic Stress Disorder

Neuroimaging studies utilize self-report scales and/or interviews to quantify duration and severity of trauma-related symptoms and relate them to brain function. **Table 1** outlines measures often employed in EEG studies to index trauma and PTSD symptoms. With respect to categorizing presence versus absence of PTSD diagnosis, researchers typically employ one or both of the following: 1) the Clinician Administered PTSD Scale (CAPS) (24, 25), considered the “gold standard” assessment of PTSD symptom severity (26); and 2) the PTSD module of Structured Clinical Interview for DSM-IV (SCID) (27). Alternatively, some studies utilize the PTSD Checklist (PCL), a brief self-report questionnaire that is highly correlated with the CAPS (*r* > .90) in place of a clinical interview; three versions assess trauma symptoms in specific (PCL-S), civilian (PCL-C), and military (PCL-M) samples (28, 29).

**Table 1:** Measures of posttraumatic stress disorder (PTSD), trauma, and resilience employed in electroencephalography (EEG) research.

Measure	Description
Clinician-Administered PTSD Scale (CAPS)¹	30 items. Measures onset/duration of past week, current month, and lifetime PTSD symptoms, subjective distress, and social functioning. Scores include three subscales: (1) re-experiencing (five items); (2) avoidance/numbing (nine items); and (3) hyperarousal (six items). CAPS-5 has been adapted for DSM-5 (20).
Structured Clinical Interview for DSM-IV (SCID), PTSD module¹	20 items. Queries current and past: (1) exposure and reactions to traumatic event; (2) three clusters of symptoms: re-experiencing, avoidance, hyperarousal; (3) symptom duration; and (4) impairment. SCID-5 has been adapted for DSM-5 (21).
Davidson Trauma Scale (DTS)²	17 items. Measures frequency of DSM-IV PTSD symptoms using a five-point scale, 0 (not at all) to 4 (everyday). A severity score can also be calculated, and total score can be computed by summing severity and frequency scores. Subscale scores can be calculated for re-experiencing/intrusion, avoidance/numbness, and hyperarousal.
Structured Interview for PTSD (SI-PTSD)¹	17 items. Indexes frequency and severity of PTSD symptoms over the past 4 weeks and during the worst period ever, and is unique in that it includes questions regarding suicidal ideation and guilt.
PTSD Checklist (PCL)²	17 items. Questionnaire assesses DSM-IV PTSD symptoms, often used to diagnose and/or track symptom changes as a function of treatment. Three versions of the PCL can be administered: civilian (PCL-C), military (PCL-M), and specific (PCL-S). Respondents use a five-point Likert scale when endorsing responses, from 1 (not at all) to 5 (extremely). Responses from all items are combined for a total severity score. PCL-5 has been adapted for DSM-5 (22).
PTSD Symptom Scale (PSS)¹^,^²	17 items. Questionnaire and interview versions assessing DSM-IV PTSD symptom severity over the past 2 weeks, as compared to other questionnaires, which measure for 1 month. Queries about a specific, single trauma and items assess re-experiencing (4), avoidance (7), and hyperarousal (6). Provides ratings for each item ranging from 0 to 3, from 0 (not at all) to 3 (five or more times). The PSS-I-5 (interview version) has been adapted to DSM-5 (23).
Childhood Trauma Questionnaire (CTQ)²	28 items. Measures the following traumatic maltreatment experiences: (1) emotional abuse; (2) sexual abuse; (3) physical abuse; (4) emotional neglect; and (5) physical neglect. Participants answer on a Likert scale ranging from 1 (never) to 5 (very often); subscores are then calculated for each type of maltreatment.
Childhood Experiences of Victimization Questionnaire (CEVQ)²	18 items. Measures various types of victimization including bullying (peer to peer), witnessing domestic violence, emotional, physical, and sexual abuse, as well as corporal punishment.
Combat Exposure Scale (CES)²	7 items. Assesses degree of stress combatants endured during wartime on a five-point frequency Likert scale (0 = no or never and 5 = more than 50 times), four-point frequency (0 = no to 4 = more than 12 times), five-point duration (1 = never to 5 = more than 6 months), or four-point degree of loss (1 = no one to 4 = more than 50%) scales.
Impact of Event Scale – Revised (IES-R)²	22 items. Measures the frequency of intrusions (eight items), avoidance (eight items), and hyperarousal (six items) associated with experienced trauma.
Mississippi Scale for Combat-Related PTSD (M-PTSD)²	35 items. Indexes symptom frequency of combat-related DSM-IV PTSD and other associated disorders (depression/suicidality/substance use) in veterans and active service military. Individuals how they feel for each item on a five-point Likert scale.
Early Life Stress Questionnaire (ELSQ)²	19 items. Queries experience of various adverse childhood events, including sexual abuse, natural disasters, major illness, domestic violence, poverty, and neglect.

¹Clinician interview. ²Self-report questionnaire. DSM-IV, Diagnostic and Statistical Manual of Mental Disorders, fourth edition; DSM-5, Diagnostic and Statistical Manual of Mental Disorders, fifth edition.

Additional measures of PTSD symptoms utilized by EEG researchers include: 1) the Davidson Trauma Scale (DTS), a brief questionnaire that possesses high concurrent validity with DSM-IV PTSD symptoms elicited by the SCID (30); 2) the Impact of Event Scale—Revised (IES-R) (31), a self-report measure highly correlated with the PCL (*r* > .80) assessing frequency of intrusion, avoidance, and hyperarousal associated with trauma (32, 33); and 3) the PTSD Symptom Scale (PSS), a self-rated scale indexing PTSD symptoms over the past 2 weeks (34). On the whole, diagnosing PTSD *via* the CAPS and is advantageous over the SCID, which is typically administered in its entirety (including multiple modules for various psychotic, mood, anxiety, and substance use disorders (SUDs), not just the PTSD module) as well as the PSS, which captures only a 2-week timeframe (when a month of symptoms are required to meet criteria for PTSD).

To address trauma encountered during childhood and adolescence, three questionnaires have been utilized in the EEG literature: 1) the Early Life Stress Questionnaire (ELSQ), which queries experience of adverse childhood events including abuse, neglect, natural disasters, major illness, adoption, poverty, and domestic violence (35); 2) the Childhood Trauma Questionnaire (CTQ), which focuses on multiple facets of abuse and neglect (36); and 3) the Childhood Experiences of Victimization Questionnaire (CEVQ), which evaluates peer bullying, corporal punishment, domestic violence, and facets of abuse (emotional, physical, and sexual) (37). In contrast to early life trauma, two scales capture traumatic symptoms suffered as a result of military service: 1) the Mississippi Scale for Combat-Related PTSD (M-PTSD) queries DSM-IV PTSD symptoms as well as comorbid substance use, suicide, and depression (38); and 2) the Combat Exposure Scale (CES) is a short questionnaire determining degree of trauma experienced in the military (from “light” to “heavy”) that is moderately correlated with M-PTSD (39).

## Navigating Electroencephalography Results Within a Clinical Framework

**Table 2** provides a summary of effect size magnitudes of studies reporting a statistically significant (most often *p* < .05) relationship between trauma/PTSD symptoms, measured categorically and/or dimensionally, and various EEG metrics. In addition to this global summary, **Tables 3****–****5** highlight particular facets of EEG study design relevant to clinical capture of trauma and PTSD. The column labeled *Trauma Group* lists the sample size for the group of interest, whereas *Trauma Type* characterizes trauma experienced within this group. Additionally the *PTSD DX* column lists measures used by each EEG study to assess trauma and/or PTSD diagnosis/symptoms, whereas *Control Group Trauma Exposure* lists the sample size for control subjects with and without trauma exposure. The *Clinical Controls* column explains whether each study excluded trauma participants with medical problems (M), neuropsychological problems (N), and psychiatric (P) problems other than PTSD. Moreover, we highlight which studies excluded participants with suicidal ideation (SUIC) as well as specific comorbid disorders such as anxiety disorders (ANX), attention-deficit hyperactivity disorder (ADHD), depression (DEP), psychosis (PSY), and SUDs. In this column, we also show which studies explicitly addressed: 1) medication effects (MED) in their trauma group; 2) time since the traumatic event occurred (TIME); and 3) sex differences within their sample (SEX). Next, the *Symptom Correlations* column highlights whether researchers correlated dimensional symptom scales with patterns of brain activity, and if so, what scale was used. Finally, the *Results* column explains findings for each study, listing significance values, and effect sizes, if they were provided or if studies included means and standard deviations, thereby enabling us to calculate effect sizes. Effect sizes reported include Cohen’s *d*, Hedges *g*, partial *η²*, *η²*, *R²*, and Spearman’s *ρ*. Asterisks next to the author name and reference (*) highlight studies that did not provide enough information for us to calculate effect sizes for at least one significant result.

**Table 2:** Effect sizes for significant electroencephalography (EEG) and event related potential (ERP) results demonstrating categorical and dimensional capture of trauma and posttraumatic stress disorder (PTSD).

	Categorical: trauma/PTSD vs control	Dimensional: correlates with trauma and/or PTSD symptoms
***EEG frequency***		
Frontal asymmetry	Small (40); Medium-large (41) Medium-large (42) Large (43)	Small-medium (42) Medium (42) Medium-large (43) Large (44)
Parietal asymmetry	Small (40) Medium (43) Large (42)	Medium (42) Large (45)
Theta power	Medium (46)	N/A
Alpha power/peak frequency	Small (46) Medium (47)	Small-medium (47)
Beta connectivity	Large (48)	Medium (48)
Gamma connectivity	Large (48)	Medium (48)
***Auditory attention and working memory***		
P2 amplitude	Medium (49) Large (50)	Medium (49) Large (51)
P2 latency	Medium-large (49)	N/A
N2 amplitude	Medium (49) Large (50)	N/A
N2 latency	Small (52) Large (50)	N/A
P3 amplitude	Medium (50) Medium (49) Medium-large (53) Large (54) Large (55) Large (56) Large (57)	Medium (58) Medium (59) Large (54) Large (50) Large (60)
P3 latency	Large (50)	N/A
Mismatch negativity (MMN) amplitude	Large (61)	Large (62)
***Auditory inhibition***		
P3 Latency	Medium-large (63)	N/A
***Visual attention and working memory***		
P2 Amplitude	Medium (64) Medium (65) Large (66) Large (67)	N/A
P3 amplitude	Small (68) Small-medium (69) Medium (70) Medium (71) Large (72) Large (73) Large (74) Large (72) Large (68)	Large (75) Large (76)
P3 latency	Medium-large (68)	Medium (76)
Late positive potential (LPP) amplitude	Medium (66) Medium (77)	Small (78, 79) Small (80) Medium (78, 79) Medium (81) Large (66) Large (75)
Contingent negative variation (CNV) amplitude	Large (82)	Small-medium (82)
N170 amplitude	Small (83) Medium (84)	Medium-large (85)
N4 amplitude	N/A	Medium (86)
P6 amplitude	Medium (77)	N/A
***Visual inhibition***		
P2 amplitude	Medium (87) Large (88)	N/A
N2 Latency	Large (89)	N/A
P3 amplitude	Large (90) Large (57) Large (88)	Large (90)
P3 latency	Large (57) Large (91)	Medium (92) Medium-large (91)
Error-related negativity (ERN) amplitude	N/A	Small (93) Small (94)
LPP amplitude	Small (95)	N/A
***Emotion regulation and reward processing***		
LPP amplitude	Medium (96)	N/A

The following benchmarks were used to categorize effect size magnitudes: 1) Cohen’s d and Hedge’s g: small = 0.2, medium = 0.5, and large = 0.8; 2) Partial η² and R²: small = .01, medium = .09, and large = .25; 3) η²: small = .01, medium = .06, and large = .14; and 4) Spearman’s ρ: small = 0.1, medium = 0.3, and large = 0.5. Studies with null findings or no effect size data are not included in this table, but are included in Tables 3–5. N/A, Not applicable.

**Table 3:** Electroencephalographic (EEG) frequency band studies of trauma and/or posttraumatic stress disorder (PTSD).

Author (Ref.)	Trauma group (T)	Trauma type	PTSD DX	Control group (C) trauma exposure?	Clinical controls	Symptom correlations	Ref/Design	Results
Bangel et al. (61)*	13	PTSD (UNKN)	CAPS	13 YC	DEP; N; P; SUIC	Y (CAPS)	AVG; two-stimulus auditory oddball (30 min)	T > YC frontal theta power (*p* < .05); T > YC right parietal alpha power suppression (*p* < .05)
Begić et al. (97)*	20	PTSD (COMBAT)	CAPS	20 NC	MED; N; P	N	LM; resting (10m, EC)	T > NC central theta (*p* < .01); T > NC frontal, central, and occipital beta (*p* < .01); T = C alpha and delta (*p* > .05)
Cowdin et al. (98)*	17	PTSD (VAR)	CAPS; SCID	13 YC	MED; N; P	N	UNKN; REM (650s, EC)	YC > T right frontal early theta power (*p* = .04) and bilateral frontal late theta power (no *p* value reported)
Curtis and Cicchetti (40)	44	Trauma (CHILD)	N/A	43 NC	MED; SEX	N	LM; Resting (8m, EC and EO)	T > NC right parietal asymmetry (*p* = .06.* partial η²* = .04); men: T > NC left frontal (糴*p* < .05, *partial η²* = .07) and occipital (*p* < .05, *partial η²* = .09) asymmetry; women: resilient NC > T right frontal asymmetry (*p* < .05, *partial η²* = .06)
Ehlers et al. (99)*	19	PTSD (VAR)	SSAGA	39 YC	N/A	N	Bipolar; resting (10–15m EC)	T > YC gamma power (*p* < .004); T > YC beta power (ᄔ*p糴* = .06)
Falconer et al. (53)	44	PTSD (UNKN)	CAPS	44 NC	N; PSY; SUD	N	LM; resting (4m, EC and EO)	T = NC delta, theta, alpha, beta power (*p* > .05)
Hostinar et al. (100)	314	Trauma (CHILD)	N/A	N/A	SEX	Y (CTQ; CES-D; STAI)	Cz; resting (6m, EC and EO)	No relationship between frontal asymmetry and CTQ *(p* = .87)
Imperatori et al. (101)*	17	PTSD (VAR)	UNKN	17 NC	MED; N; P	N	LM; resting (5m, EC)	T > NC frontal (*p* < .05) and parietal (*p* < .05) theta activity; T > NC parietal alpha connectivity (*p* < .05)
Kemp et al. (44)	14	PTSD (UNKN)	CAPS	15 NC; 15 MDD+	DEP; N	Y (CAPS, DASS)	LM; resting (2m, EC)	T = NC frontal asymmetry (*p* > .05); T: higher CAPS linked to greater right frontal asymmetry (*p* = .02, *R²* = .38)
Lee et al. (48)	33	PTSD (ACCID)	SCID	30 NC	N/A	Y (DTS; SIP; HAM-D)	Cz; resting (5m, EC)	NC > T frontocentral beta (*p* = .0002, *d* = 1.09) and gamma (*p* = .003, *d* = 0.91) connectivity; T: higher DTS-total (*p* = .003, *R²* = .25), SIP arousal (*p* = .03, *R²* = .14), and HAMD (*p* = .03, *R²* = .14) linked to lower connectivity
McFarlane et al. (35)*	214	Trauma (ELS)	N/A	193 NC	M; N; P; SEX	Y (ELSQ)	LM; resting (4m, EC and EO)	NC > T EO beta (*p* = .007), theta (*p* < .001), alpha (*p* = .01), and delta (*p* < .001) power, and EC beta, theta, alpha and delta power (all *p* < .001)
Metzger et al. (45)	29	PTSD (COMBAT)	CAPS; SCID	13 YC	MED; PSY	Y (CAPS; SCL-90R depression)	LM; resting (6m, EC and EO)	CAPS-arousal, depression, and their interaction (*p* = .01, *R²* = .25) linked to greater right parietal asymmetry
Meyer et al. (43)	24	PTSD (VAR)	SCID	15 YC; 15 NC	N; PSY; SUD; TIME	Y (PCL; PSS; RIQ; BDI-II)	LM; resting (8m, EC and EO); neutral image (2m); positive image (2m); negative image (2m); trauma image (2m)	Resting: T and YC > NC for left frontal asymmetry (*p* < .001, *partial η²* = .30); T > NC for left parietal asymmetry (*p* = .04, *partial η²* = .12); neutral: YC > T left frontal asymmetry (*p* < .03, partial *η*² = .13); negative: YC > T and NC left frontal asymmetry (*p* < .03, partial *η*² = .25); negative: higher right frontal asymmetry linked to higher PCL, PSS, RIQ, and BDI-II symptoms (all *p* < .01, *R²* range: .18–.32); trauma: higher right frontal asymmetry linked to greater emotional intensity/physical reaction to image (both *p* < .001, *R²* = .24–.25)
Miskovic et al. (41)	38	Trauma (CHILD)	UNKN	24 NC	MED	N	AVG; resting (2m EC and EO) 6 months apart	T > NC right frontal asymmetry at time 1 (*p* = .01, T > NC right frontal asymmetry at ² = .26) and time 2 (*p* = .04, T > NC right frontal asymmetry at ² = .08)
Rabe, Beauducel et al. (42)	43	PTSD (ACCID)	CAPS; SCID	21 YC; 23 NC	MED; N; PSY; SEX; SUD; TIME	Y (CAPS; BDI)	LM; resting (8m, EC and EO); neutral image (1m); positive image (1m); negative image (1m); trauma image (1m)	Trauma-related right frontal asymmetry for full T (*p* < .09, *η*² = .13) and partial T (*p* <.08, *η*² = .15); trauma-related right parietal asymmetry for full T (*p* < .05, *η*² = .19) and partial T (*p* < .05, *η*² = .24); YC: trauma-related left frontal asymmetry (*p* < .01*, η*² = .36); higher CAPs linked to greater trauma-related right frontal and parietal asymmetry (all *p* < .05; *R²* range: .08–.23)
Rabe, Zöllner et al. (102)	45	PTSD (ACCID)	CAPS; SCID	37 YC	MED; N; PSY; SUD; TIME	Y (PTGI)	LM; resting (8m, EC and EO)	Higher left frontocentral asymmetry linked to greater PTGI subscales (all *p* < .05, *R²* range: .05–.17)
Shankman et al. (103)	32	PTSD (ACCID, ASSAULT)	CAPS	42 NC	SEX; TIME	Y (CAPS; DASS)	AVG; resting (4m, EC and EO)	T = NC for frontal, central, and parietal asymmetry (all *p* > .05)
Tang et al. (104)*	43	Trauma (CHILD)	KSADS; CTQ; CEVQ	N/A	DEP; MED	Y (CTQ)	AVG; resting (2m, EC and EO) in three sessions over 2 years	Right frontal asymmetry interacted with trauma to predict future PTSD outcome (*p* = .03)
Todder et al. (105)*	10	PTSD (UNKN)	UNKN	10 NC	N; PSY; SUD; TIME	N	AVG and LM; resting (3m, EC)	T = NC absolute theta power (糴*pᄔ* > .05); using LORETA source localization software: (1) NC > T for right temporal low theta, and 2) NC > T for bilateral frontal high theta (no *p* values reported)
Veltmeyer et al. (46)	34	PTSD (VAR)	CAPS	136 NC	N; P; TIME	Y (CAPS)	LM; resting (2m, EO)	NC > T low alpha power (糴*pᄔ* = .09, *d* = .47); NC > T theta power (ᄔ*p糴* =.08, *d* = .53); NC > T high alpha power (糴*pᄔ* = .04, d = .34) and theta/alpha ratio (ᄔ*p糴* = .05, d = .19)
Wahbeh and Oken (47)	57	PTSD (COMBAT)	CAPS; SCID; CES	29 YC	N; PSY; SUD	Y (CAPS; PCL)	AVG; resting (5m, EC)	T = YC for frontal, central, parietal asymmetry (all *p* > .10); T > YC for peak alpha frequency (*p* < .01, *d* = .57); T : higher global peak frequency linked to higher CAPS total/subscale and PCL scores (all *p* < .05, *ρ* range: .22–.33)

*Effect sizes for at least one significant effect unable to be computed based on article information. ACC, traumatic accident; AVG, average reference; BD, Beck Depression Inventory; CAPS, Clinician-Administered PTSD Scale; CES, Combat Exposure Scale; CES-D, Center for Epidemiological Studies Depression Scale; CEVQ, Childhood Experiences of Victimization Questionnaire; CHILD, child abuse; CTQ, Childhood Trauma Questionnaire; DASS, Depression Anxiety Stress Scales; DEP, excluded for depression; DTS, Davidson Trauma Scale; DX, diagnosis; EC, eyes-closed recording; ELS, early life stress; ELSQ, Early Life Stress Questionnaire; EO, eyes-open recording; HAMD, Hamilton Rating Inventory for Depression; LM, linked mastoids; M, excluded for medical problems; MDD, major depressive disorder; MED, excluded for medication; N, excluded for neurological problems; N/A, not applicable; NC, trauma-no control group; ND, natural disaster; P, excluded for psychiatric diagnoses; PCL, PTSD Checklist; PGTI, Posttraumatic Growth Inventory; PSS, PTSD Symptom Scale; PSY, excluded for psychosis; Ref, reference montage; REM, rapid eye movement; RIQ, Response to Intrusions Questionnaire; SCID, Structured Clinical Interview for DSM-IV; SCL-90, Symptom Checklist 90; SEX, explicitly examined sex differences; SIP, Structured Interview for PTSD; STAI, State Trait Anxiety Inventory; SUD, excluded for substance use disorders; SUIC, excluded for suicidal ideation; T, trauma group; TIME, reported time since trauma; UNKN, unknown; VAR, various traumas; YC, trauma-yes control group.

**Table 4:** Auditory event related potential (ERP) studies of trauma and/or posttraumatic stress disorder (PTSD).

Author (Ref.)	Trauma group (T)	Trauma type	PTSD DX	Control group (C) trauma exposure?	Clinical controls	Symptom correlations	Results
***Auditory attention and working memory***							
Araki et al. (54)	8	PTSD (TERROR)	CAPS	13 YC	MED; N; P; TIME	Y (CAPS; STAI; IES-R)	YC > T parietal target P3 amplitude (*p* = .04, *d* = .81); YC = T for P3 latency (*p* = .49); within T, lower P3 amplitude linked to higher CAPS avoidance (*p* = .04, *ρ* = .85).
Bae et al. (58)*	30	PTSD (ACCID)	SCID	33 NC	N; P	Y (SCID; DTS)	NC > T P3 amplitude (*p* < .01); T = NC P3 latency (*p* > .15); using P3 amplitude source analysis (LORETA): (1) higher re-experiencing linked to parietal P3 (*p* < .05, *R²* range: .14–.24), (2) higher avoidance/numbing linked to higher temporal/parietal P3 and lower frontal P3 (*p* < .05, *R²* range: .13–.17), and (3) higher hyperarousal linked to frontal and temporal/parietal P3 (*p* < .05, *R²* range: .14–.20)
Bangel et al. (61)	13	PTSD	CAPS	13 YC	DEP; N; P; SUIC	Y (CAPS)	T > YC MMN amplitude to deviants (*p* < .001, *n²* = .63)
Blomhoff et al. (106)*	11	PTSD (FIRE)	CAPS; IES-R	9 YC	N; PSY; SUD; TIME	Y (CAPS)	T > YC positive/negative P2–P3a amplitudes linked to arousal (*p* < .01) and avoidance (*p* < .001)
Boudarene & Timsit-Berthier (55)	19	PTSD (UNKN)	UNKN	17 YC; 18 NC	TIME	N	YC > T frontal P3a (*p* = .0004, *d* = .75) and P3b (*p* = .0001, *d* = .78) amplitudes; NC > T frontal P3a (*p* = .0004, *d* = 1.18) and P3b (*p* = .0001, *d* = 1.33) amplitudes
Charles et al. (56)	16	PTSD (ASSAULT)	SADS	10 NC	M; MED; P; SEX; TIME	N	NC > T central target P3 amplitude (糴*pᄔ* < .001, *d* = 2.70); T = NC P3 latency (*p* = .46)
Falconer et al. (53)	44	PTSD (UNKN)	CAPS	44 NC	N; PSY; SUD	N	NC > T frontal (*d* = .53), central (*d* = 3.40), and parietal (*d* = .70) P3 target amplitude (*p* < .02)
Felmingham et al. (50)	17	PTSD (ASSAULT, ACCID)	CAPS	17 NC	DEP; MED; N; PANIC; PSY; SUD; TIME	Y (CAPS)	NC > T P2 amplitude (糴*pᄔ* < .01, *d* = 1.17); T > NC N2 amplitude (ᄔ*p糴* < .05, *d* = 1.24); T > NC frontal N2 latency (糴*pᄔ* < .05, *d* = 1.20); NC > T P3 amplitude (ᄔ*p糴* < .05, *d* = .72); T > NC parietal P3 latency (ᄔ*p糴* < .05, *d* = .84); greater numbing linked to lower P3 amplitude (*p* = .01, *R²* = .35)
Felmingham et al. (107)*	17	PTSD (ASSAULT, ACCID)	CAPS	12 YC (ASD+); 13 NC (ASD−)	DEP; MED; N; PANIC; PSY; SUD	N	ASD+ > parietal P3b target amplitude than PTSD+ and ASD− (*p* < .001)
Galletly et al. (108)*	18	PTSD (VAR)	DIS	18 NC	MED; N; SUD	Y (STAI)	T > NC N2 target latency (*p* < .05); NC > T P3 target amplitude (*p* < .05)
Ge et al. (109)*	13	PTSD (ND)	PCL-C	14 YC	MED; N; P; SUD	N	T > YC MMN amplitude (*p* < .05)
Hall et al. (110)	12	PTSD (COMBAT)	CAPS; SCID; M-PTSD	12 MZ twins NC; 23 MZ twins YC; 23 MZ twins NC	M	N	T = YC/NC P3b amplitude (*p* > .05)
Kimble et al. (111)	24	PTSD (COMBAT)	CAPS; CES	15 YC	N; SUD	N	T = YC P3a/P3b amplitude (*p* > .05)
Kimura et al. (59)	29	Trauma (ND)	N/A	N/A	P; TIME	Y (IES-R)	Higher trauma central P3a amplitude linked to higher IES-R hyperarousal (*p* = .03, *ρ* = .40) but not total (*p* = .07), intrusions (*p* = .24), or avoidance (*p* = .19).
Kimura et al. (60)	13	Trauma (ND)	N/A	N/A	N/A	Y (IES-R)	No links between P3a amplitude and IES-R total, intrusions, avoidance, or hyperarousal (all *p* > .30); lower P3b amplitude associated with higher IES-R intrusions (*p* < .05, *ρ* = -.56) but not any other IES-R scores (all *p* >.13).
Lamprecht et al. (112)*	10	PTSD (VAR)	DSM-IV; IES	10 NC	MED; SUD; TIME	Y (IES)	T > NC for N1 amplitude (*p* < .02); T = NC for P2 (*p* = .33), N2 (*p* = .27), and P3b (*p* = .58) amplitudes; T showed novel P3a amplitude reduction post- as compared to pre-treatment (*p* < .03)
Lewine et al. (51)*	31	PTSD (COMBAT)	SCID; CAPS; M-PTSD	38 NC; 10 MDD+; 8 AUD+	M; N	Y (CAPS; M-PTSD; HAM-D)	T = NC for N1 amplitude (*p* = .26); NC > T P2 amplitude to high intensity tones (*p* < .01, no effect size able to be computed); within T, higher amplitude linked to higher CAPs/M-PTSD (both *p* = .03, *R²* = .52) and higher HAM-D (*p* = .04, *R²* = .49) scores
McFarlane et al. (35)*	214	Trauma (ELS)	N/A	193 NC	M; N; P; SEX	Y (ELSQ)	NC > T for frontal N2 target amplitude (*p* = .006)
McPherson et al. (113)*	60	PTSD (CPA, CSA)	DICA	81 YC	SEX	Y (DICA)	T > YC P2-N2 amplitude to high intensity tones (*p* < .05); P2-N2 amplitude linked to re-experiencing symptoms (*p* = .02)
Menning et al. (114)*	10	PTSD (VAR)	SCID	14 NC	TIME	N	T > NC MMN amplitude (*p* < .05); T = NC for N1-P2 amplitude (*p* = .22)
Metzger et al. (57)	34	PTSD (COMBAT, CSA)	UNKN	18 YC	PANIC; SEX	N	YC > T target P3b amplitudes (*p* < .05, *d* = 1.30); in women, YC > T distractor P3a amplitudes (*p* = .04, *d* = .91)
Metzger et al. (49)	29	PTSD (COMBAT)	SCID; CAPS; PCL-M; M-PTSD; IES-R	37 YC	PSY; MED	Y (CAPS; PCL-M)	T > YC target P3b amplitude (*p* < .05, *d* = .50); T > YC P2 target amplitude (*p* = .007, *d* = .68); T > YC P2 frontal and parietal non-target amplitude (*p* =.03, *d* = .46 and .54); T > YC P2 target latency (*p* = .03, *d* = .55); T > YC P2 non-target latency (*p* < .001, *d* = .91); YC > T frontal N2 target amplitude (*p* = .03, *d* = .52); higher P2 amplitude linked to greater CAPs total, re-experiencing, avoidance/numbing, and arousal (*p* ≤ .05; *R²* range: .08–.10) and IES-R (*p* = .05, *R²* = .08).
Metzger et al. (52)	37	PTSD (COMBAT)	CAPS; M-PTSD; CSS	37 MZ twins NC; 47 MZ twins YC; 48 MZ twins NC	PSY; MED	N	T > YC/NC N2 latency (*p* = .04, *d* = .45); T = YC/NC for P3b amplitude *(p* > .05)
Morgan & Grillon (62)*	13	PTSD (ASSAULT)	SCID	16 NC	M; MED; SUD	Y (M-PTSD; STAI)	T > NC MMN target amplitude (*p* < .05) and N2 target amplitude (*p* < .02); greater frontal MMN amplitude linked to greater PTSD severity (*p* < .05, *R²* = .36)
Neylan et al. (115)	25	PTSD (COMBAT)	CAPS; SCID	15 YC	N; PANIC; PSY	Y	T = YC P3a/P3b amplitude (*p* > .27) and latency (*p* > .05)
***Auditory inhibition***							
Schaefer & Nooner (63)	12	Trauma (VAR)	N/A	26 NC	N/A	Y (TSC-40) and depression	T > NC frontal Go P3 latency (trauma + depression) (*p* = .003, *d* = 1.14); NC > T central/parietal Go P3 latency (trauma + depression) (*p* < .02, *d* = .74 and.81)

**Table 5:** Visual event related potential (ERP) studies of trauma and/or posttraumatic stress disorder (PTSD).

Author (Ref.)	Trauma group (T)	Trauma type	PTSD DX	Control group (C) trauma exposure?	Clinical controls	Symptom correlations	Results
***Visual attention and working memory***							
Attias et al. (116)*	20	PTSD (COMBAT)	UNKN	20 YC	MED; N; P	N	T > YC combat P3a amplitudes (*p* < .05); T > YC target/combat N2 amplitudes (*p* < .01); T > YC P3 target latency (*p* < .02); no group differences in P2 amplitudes
Bleich et al. (72)	20	PTSD (COMBAT)	UNKN	20 YC	MED; N; P;	N	T > YC target, combat, and irrelevant non-target P3a/P3b amplitude (*p* < .001; *d* = 1.14)
Chu et al. (83)	489	Trauma (CHILD)	N/A	N/A	M; P; SEX	Y (ELSQ)	N170 amplitude for angry > happy faces linked to child trauma in right temporal region, such that high trauma was associated with less N170 differentiation between these faces than low trauma (*p* < .04, partial *η*² = 2.2% variance); N170 amplitude for angry > happy faces linked to adult trauma in left temporal region, wherein high T was associated with less N170 differentiation between these faces than low trauma (糴*pᄔ* < .01, partial *η*² = 2.1% variance)
Chu et al. (85)	72	Trauma/ PTSD (CHILD)	CAPS; MINI	N/A	PSY; SEX; SUD	Y (CAPS; ELSQ)	Lower N170 amplitudes for fear > happy faces linked to higher child trauma in left temporal region (*p* = .002; *R²* change = .15); higher PTSD avoidance linked to lower N170 amplitude to happy faces (*p* = .004, *partial η²* = .30).
DiGangi et al. (78)	73	Trauma (VET)	PCL-M; MINI	N/A	M; PSY; SUIC	Y (PCL-M)	Higher PTSD symptoms linked to smaller LPP amplitude to angry faces (*p* = .04. *R²* = .09), but not happy or fear faces (*p* > .05)
DiGangi et al. (79)	47	Trauma (VET)	CAPS; MINI;	N/A	M; PSY; SUIC	Y (CAPS)	Within T with greater perseverative errors, higher PTSD symptoms linked to larger angry face LPP amplitude, but in those with smaller errors, higher PTSD symptoms linked to smaller LPP amplitude to angry faces (*p* = .02, *R²* = .07)
Duan et al. (82)	28	PTSD (ND)	PCL	30 YC	MED; N; SUD; TIME	Y (PCL)	T > YC CNV amplitude (*p* < .05, *d* = 2.94); greater CNV amplitude linked to higher PCL total (*p* < .05, *R²* = .07) and re-experiencing (*p* < .05, *R²* = .09)
Ehlers et al. (99)*	19	PTSD (VAR)	SSAGA	39 YC	N/A	N	T > YC happy central (*p* < .04) and right frontal (*p* < .05) P3 latency
Gilmore et al. (70)	33 PTSD, 19 TBI, 41 TBI-PTSD	PTSD (COMBAT)	CAPS; SCID	31 TBI-YC	DEP; M; PSY; SUD	Y (CAPS)	No group differences in P1, N1, P2, or N2 amplitudes (all *p* > .05); YC > T parietal P3b target amplitude (*p* < .002, *partial η²* = .12); no significant correlations between CAPS total/subscales and P3b amplitude (all *p* > .12)
Grasso & Simons (117)	19	PTSD (CHILD)	KSADS	19 NC	PSY; SUD; SUIC; TIME	N	T = NC LPP amplitude to negative pictures (*p* > .05)
Honzel et al. (118)*	17	PTSD + TBI (COMBAT)	PCL-M	16 YC	P	N	Single task: T =YC on P3 amplitude (*p* > .05); dual task: YC old probes > P3 amplitude than new probes, whereas T old probes = new probes for P3 amplitude (*p* = .001)
Kessel et al. (119)	37	Trauma (ND)	N/A	40 YC	M; TIME	Y (hurricane stress)	YC lower post- than pre-trauma negative LPP amplitude (*p* = .007, *d* = .61); within T, negative LPP amplitude did not change pre- and post-trauma (*p* = .93)
Kimble et al. (120)*	22	PTSD (VAR)	SCID; CAPS	35 YC	N; PSY; SUD	N	YC > T N4 amplitude to threatening versus expected sentence endings (*p* < .01); YC = T for N4 latency (*p* > .05)
Kimble et al. (86)	18	PTSD (VAR)	PSS; TES	21 YC	N; MED; SUD	Y (PTCI)	T = YC N4 amplitude to positive, negative, and incongruent sentence endings (*p* > .05; *η*² = .01); greater PTCI negative cognitions about the world linked to larger N4 amplitudes to negative sentence endings (*p* < .01; *R²* = .18)
Klimova et al. (121)*	39	PTSD (VAR)	CAPS	N/A	N; SUD; TIME	Y (CADSS)	Low dissociating T > high dissociating T P2 amplitude for consciously presented happy faces (*p* = .04)
Kounios et al. (122)*	8	PTSD (COMBAT)	UNKN	8 YC	N/A	N	T > YC central P3 amplitude; T > YC LPP amplitude (no* p* values provided)
Kujawa et al. (80)	260	Trauma (ND)	N/A	N/A	M; TIME	Y (hurricane stress; CBCL)	Higher stress interacted with greater LPP amplitude to unpleasant images to predict greater externalizing symptoms (*p* < .001, *R²* change = .07), whereas higher stress and larger LPP amplitude to unpleasant images (*p* < .04, *R²* change = .05) predicted higher internalizing symptoms
Lobo et al. (81)	43	Trauma (VAR)	PCL-C	N/A	MED; N; P;	Y (PCL-C)	Higher trauma symptoms linked to greater unpleasant minus neutral LPP amplitude (*p* < .01, *R²* = .22)
MacNamara et al. (66)	19	PTSD (COMBAT)	SCID; CAPS; PCL-M; CES	14 YC	M; MED; N; PSY	Y (CAPS)	YC > T P2 amplitudes for emotional faces (*p* < .01; *partial η²* = .25); YC > T LPP amplitudes to angry faces (*p* < .01, *partial η²* = .12); smaller LPP to fearful faces linked to greater CAPs intrusions (*p* < .02, *R²* = .31)
Saar-Ashkenazy et al. (77)	14	PTSD (VAR)	CAPS	14 NC	N; P	N	T = NC P300 amplitude (*p* >.05); NC > T neutral P600 amplitude (*p* = .05, *partial η²* = .22); a group x region x emotion interaction (*partial η²* = .16) showed that T > NC positive left frontocentral LPP amplitude (*p* < .01) and T > NC neutral left parieto-occipital LPP amplitude (*p* < .05)
Shah et al. (71)	18	PTSD (VAR)	PCL; IES	18 NC	N; PSY	N	NC > T P3 amplitude to neutral-neutral dot probe condition (*p* < .05, *d* = .71)
Shu, Onton, Prabhakar et al. (123)*	16 + TBI	PTSD (COMBAT)	SCID; CAPS	16 + TBI YC	ADHD; M; PSY; SUD	Y (CAPS)	T > YC greater N170/P2, N2/P3, and LPP amplitudes (all *p* < .05); N2/P3 amplitude correlated with CAPS total (*p* < .01)
Sokhadze et al. (124)*	10 + CD	PTSD (UNKN)	SCID; PSS	12 CD+ NC; 9 CD- NC	N/A	N	T > CD− P3a amplitude to trauma (*p* < .01); T > CD+ P3a trauma latency (*p* = .01)
Stanford et al. (68)	10	PTSD (COMBAT)	SCID; PCL-M; CES	10 YC	ADHD; M; N; PSY; SUD	N	YC > T right parietal target P3 amplitude (*p* = .03, *d* = .45); T > YC frontal combat P3 amplitude (*p* < .05, *d* = .90); YC > T P3 central and parietal target latency (*p* < .03, *d* = .56 and.81).
Tillman et al. (73)	22	Trauma (COMBAT)	SCID; CAPS; M-PTSD	8 YC	N/A	Y (M-PTSD)	T = YC for P3a amplitude (*p* = .80); YC > T target P3b amplitude (*p* = .001, *η*² = .38); lower target P3b amplitude linked to higher hyperarousal (*p* = .004, *R²* = .63)
Trujillo et al. (84)	30	Trauma (COMBAT)	N/A	20 NC	DEP; MED; N; SUD	N	T > NC N170 amplitude to faces than words (*p* < .001, *d* = .60)
Tso et al. (75)	31	Trauma (TERROR)	N/A	N/A	M; MED; P; TIME	Y (IES-R)	Higher avoidance linked to lower P3 and LPP amplitude to attack than neutral stimuli (both *p* < .01, *R²* = .24 and .25)
Veltmeyer et al. (69)	34	PTSD (VAR)	CAPS; PCL	136 NC	ANX; PSY; SUD; TIME	Y (CAPS)	NC > T left temporal and midline centroparietal P3 amplitude (both *p* < .01, *d* = .29 and .51); no links between CAPS and ERPs (*p* > .05)
Wang et al. (74)	65	Trauma (COMBAT)	CAPS; PCL-M	N/A	N; PSY; SUIC	N	Lower target P3b amplitude predicted future PTSD conversion (*p* < .05, *d* range: 1.11–1.36), but no effect emerged for P3b latency (*p* > .05)
Wang et al. (76)	30	Trauma (COMBAT)	CAPS; PCL-M	N/A	DEP; N	Y (CAPS; PCL-M)	No subjects had PTSD at baseline. Increased target P3b amplitude (*p* < .0001, *R²* = .52) and latency (*p* = .02, *R²* = .18) change was associated with greater PTSD and depression symptom reductions from baseline to follow-up
Wessa et al. (67)	7	PTSD (ACCID)	SCID; CAPS	7 YC; 7 NC	N	Y (PDS)	NC > T P2 amplitude for neutral (*d* = 1.19), positive (*d* = 1.30), accident (*d* = 1.32) pictures (all *p* < .05); YC > T P2 amplitude for neutral (*d* = 1.56), positive (*d* = 1.43), accident (*d* = 2.85) pictures (all *p* < .05); no LPP differences.
Wessa et al. (125)*	16	PTSD (UNKN)	SCID; CAPS	15 YC; 16 NC	N	N	T > NC P3/LPP amplitude to trauma questions (*p* < .05); NC > T and YC frontal CNV trauma amplitude (*p* < .04)
Yun et al. (126)	12	Trauma (ND)	PTSD-SS	12 YC	N; P	N	T: earthquake-related stimuli elicited larger P3 amplitude than unrelated stimuli (*p* < .06, *d* = .74 within-group), pattern not seen in YC; P3 localized to parahippocampal gyrus
Zhang et al. (64)	13	Trauma (ND)	PTSD-SS	13 NC	N/A	N	T > NC P2 amplitude (*p* = .03, *partial η²* = .17)
Zhang et al. (127)*	13	Trauma (ND)	PTSD-SS	13 NC	N/A	N	NC > T P2 latency to trauma stimuli (*p* <.01); T > NC P2 frontal/central amplitude to trauma stimuli (*p* < .05); T > NC P3 amplitude to trauma stimuli (*p* < .001); T > NC LPP amplitude to trauma stimuli (*p* < .05)
Zuj et al. (65)	21	PTSD (COMBAT)	PCL-M	21 YC	N	N	T > YC P1 (*p* = .02, *partial η²* = .13) and P2 (*p* = .001, *partial η²* = .13) amplitude to angry faces pre- to post-deployment; T = YC for N170, N2, and P3 amplitude (all *p* > .05)
***Visual inhibition***							
Chen et al. (128)*	11	PTSD (ND)	PSS	11 YC	M; MED; P; TIME	N	YC > T P2 amplitude (*p* = .03); YC: greater P3 amplitude for trauma than non-trauma (*p* = .003) but T P3 conditions did not differ (*p* > .05)
Covey et al. (90)	14	Trauma (POLICE)	CAPS; PCL-C	11 NC	M; MED; P	Y (CAPS)	T > NC P3 amplitude across conditions (*p* < .01, *partial η²* = .26); T = NC for P3 latency and N2 nogo amplitude (*p* > .05); higher frontal nogo P3 amplitude linked to greater CAPS lifetime and total scores (all *p* < .06, *R²* range: .27–.48)
Cui et al. (87)	19	PTSD (VIOLENT)	PCL-C	15 NC	M; P	N	NC > T P2 amplitude across conditions (*p* = .02, *d* = .66)
Gorka et al. (129)	43	PTSD (VET)	CAPS; SCID	24 YC	M; N; PSY	Y (CES, CAPS)	T = YC in ERN amplitude (*p* > .05); within T, AUD+ had greater ERN amplitudes than PTSD alone (*p* < .01, *Hedge’s g* = .34); CES and CAPS were unrelated to ERN amplitude (*p* > .05)
Khan et al. (93)	67	Trauma (VET)	MINI; CAPS; CES	N/A	M; N; PSY; SUD	Y (CES; DRRI-2)	Greater combat exposure (DRRI-2) linked to larger ERN amplitude above and beyond anxiety and PTSD symptoms (*p* = .01, *R² = .*10); no relationship between ERN amplitude and CES (*p* = .26, *R² = .*02).
Lackner et al. (130)*	43	Trauma (CHILD)	CTES	N/A	N/A	Y (CTES)	High T showed larger correct related negativity (CRN) minus ERN difference compared to low and medium T (*p* = .10)
Lieberman et al. (131)*	47	Trauma (VAR)	SCID	N/A	MED; N; PSY; SEX	Y (SCID)	Higher ERN amplitude linked to greater PTSD hyperarousal (*p* = .03) and avoidance (*p* = .06) symptoms
Melara et al. (95)	16	PTSD	SCID; CAPS	14 YC; 15 NC	DEP; PSY; SUD; SUIC		T > YC/NC threat-related distractor frontocentral positivity 600–900 ms (*p* < .05, *η*² = .05) localized to posterior cingulate cortex
Metzger et al. (57)	9	PTSD (VAR)	SCID	10 NC	MED	N	T > NC P3 latency across conditions (*p* = .03, *d* = 1.00); NC > T P3 amplitude across conditions (*p* = .0002, *d* = 1.54)
Meyer et al. (94)	223	Trauma (ND)	N/A	N/A	M	Y (hurricane trauma; CBCL)	Children with higher stress and higher ERN amplitude show greater internalizing symptoms post-trauma (*p* < .01, *R²* = .06)
Rabinak et al. (132)	16	PTSD (COMBAT)	SCID; CAPS; PCL-M; CES	18 YC; 16 NC	MED	N	T = YC/NC for ERN amplitude (*p* > .05)
Qiu et al. (133)	12	Trauma (ND)	N/A	12 YC	N; P;	N	Within T, N400/600 to incongruent than congruent (*p* < .05, within-group *d* = .57), localized to right prefrontal regions
Shucard et al. (91)*	23	PTSD (COMBAT)	CAPS; DTS; SCID	13 NC	N; SUD	Y (CAPS)	T = NC for go/nogo P3 amplitude (*p* > .05); T > NC nogo P3 latency (*p* = .001, *d* = 1.07); T > NC distractor frontal P3 amplitude (*p* = .001; effect size not able to be calculated); longer P3 go and nogo latency linked to higher CAPS hyperarousal (both *p* < .05, *R²* = .19, .21); longer frontal, central, and parietal distractor latency linked to greater re-experiencing (all *p* < .01, *R² range* = .31–.46)
Shu, Onton, O’Connell et al. (134)*	17 + TBI	PTSD (COMBAT)	SCID; CAPS	15 + TBI YC	ADHD; M; PSY; SUD	Y (CAPS)	T > YC N2 amplitude (*p* < .001); no group differences in P3 amplitude (*p* > .05); greater N2 amplitude linked to worse CAPS total, avoidance/numbing, and hyperarousal (*p* < .001); N2 localized to dorsal anterior cingulate
Swick et al. (135)	14 + TBI	PTSD (COMBAT)	SCID; PCL-M	5 YC; 9 NC	SUD	Y (PCL-M, BDI)	T = YC/NC on ERN amplitude (*p* > .05); ERN not correlated with PTSD or depression symptoms within T (all *p* > .25)
Wei et al. (88)	14	Trauma (ND)	PTSD-SS	14 NC	N; P; TIME	N	T: P2 and P amplitudes greater for positive than negative words (both *p* < .05, within-group *d* = 1.14 and 1.83), latter localized to parahippocampal gyrus/cuneus
Wu et al. (89)	16	PTSD (ND)	PCL-C	9 YC	MED; N; P; SUD	N	YC > T nogo N2 latency (ᄔ*p糴* = .02, *d* = 1.03); YC = T P3 amplitude/latency (*p* > .05)
Wu et al. (92)	54	Trauma (ND)	PCL-S	N/A	MED; N; P; SUD	Y (PCL-S)	Greater PTSD avoidance symptoms linked to longer P3 nogo latency (*p* = .01, *R²* = .13)
***Emotion regulation and reward processing***							
Fitzgerald et al. (96)	25	PTSD (COMBAT)	SCID; MINI; CAPS; PCL-M; CES	25 YC	M; N; PSY	N	T = YC for LPP before or during reappraisal (*p* > .05); T showed lower LPP increases than YC for maintain (*p* = .02, *partial η²* = .09)
Fitzgerald et al. (136)*	54	Trauma/ PTSD (COMBAT)	MINI; CAPS; CES	N/A	M; N; PSY; SUIC	Y (CAPS)	Smaller change in LPP amplitude during reappraisal was associated with greater PTSD re-experiencing (*p* < .01) and avoidance (*p* = .03); smaller change in LPP amplitude during emotion experience was linked to lower PTSD avoidance symptoms over time (*p* < .01)
Li et al. (137)*	18	Trauma (ND)	N/A	22 NC	TIME	N	T > NC for P2 and P3 amplitudes (both *p* < .05)
Pechtel et al. (138)*	15 past MDD+	Trauma (CSA)	N/A	DC: 16 past MDD+; NC: 18 past MDD-	M; MED; N; PSY; SUD	N	NC = T ERN amplitude (*p* = .10); T > DC subgenual ACC for correct novel trials

*Effect sizes for at least one significant effect unable to be computed based on article information. ACCID, accidents; ADHD, excluded for attention deficit hyperactivity disorder; ANX, excluded for anxiety disorder; AUD, alcohol use disorder; BDI, Beck Depression Inventory; CADSS, Clinician Administered Dissociative States Scale; CAPS, Clinician Administered PTSD Scale; CBCL, Child Behavior Checklist; CD, cocaine dependence; CES, Combat Exposure Scale; CHILD, child abuse; CNV, contingent negative variation event related potential; CSA, childhood sexual abuse; CTES, Childhood Trust Events Survey; DTS, Davidson Trauma Scale; DC, depressed control group; DEP, excluded for depression; DRRI-2, Deployment Risk and Resilience Inventory; DX, diagnosis; IES-R, Impact of Events Scale Revised; ELSQ, Early Life Stress Questionnaire; ERN, error-related negativity event related potential; KSADS, Schedule for Affective Disorders and Schizophrenia, Child Version; LPP, late positive event related potential; M, excluded for medical problems; M-PTSD, Mississippi Scale for Combat-Related PTSD; MDD, major depressive disorder; MED, excluded for medication; MINI, Mini International Psychiatric Interview; N, excluded for neurological problems; N/A, not applicable; N170, N170 event related potential; N2, N200 event related potential; N4, N400 event related potential; NC, trauma-no control group; ND, natural disaster; P, excluded for psychiatric diagnoses; P1, P100 event related potential; P2, P200 event related potential; P3, P300 event related potential; P3a, frontocentral P300; P3b, centroparietal P300; PCL, PTSD Checklist; PCL-C, PTSD Checklist, Civilian Version; PCL-M, PTSD Checklist, Military Version; PCL-S, PTSD Checklist, Specific Version; PDS, Posttraumatic Stress Diagnostic Scale; PSS, Posttraumatic Stress Scale; PSY, excluded for psychosis; PTCI, Posttraumatic Cognitions Inventory; PTSD-SS, PTSD Self Rating Scale; SCID, Structured Clinical Interview for DSM-IV; SEX, explicitly examined sex differences; SSAGA, Semi-Structured Assessment for the Genetics of Alcoholism; SUIC, excluded for suicidal ideation; SUD, excluded for substance use disorders; TBI, traumatic brain injury; TES, Traumatic Events Scale; TIME, reported time since trauma; TERROR, terrorist attack; UNKN, unknown; VAR, various traumas; VET, veterans; YC, trauma-yes control group.

## Electroencephalography Measured in Frequency and Time: What Is the Difference?

**Figure 1:**
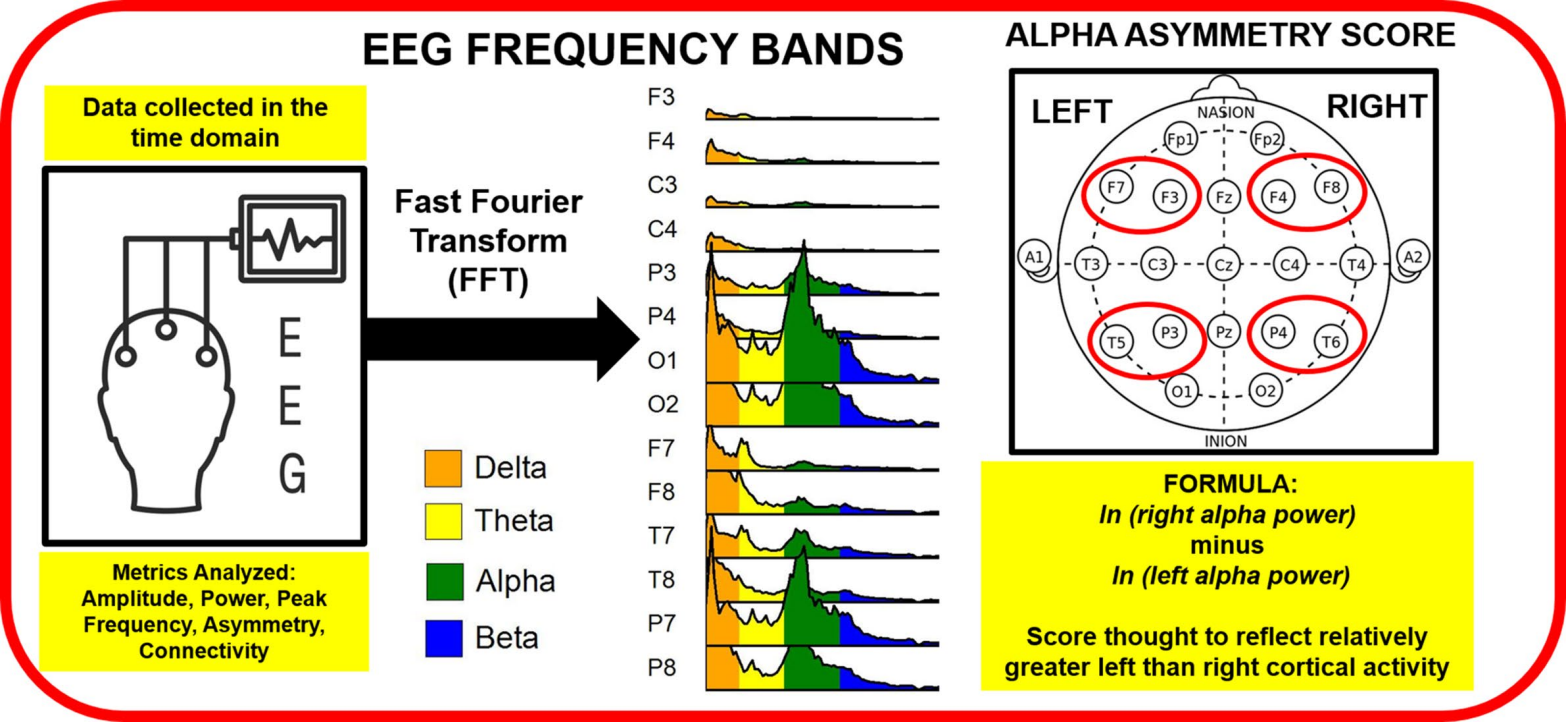
Electroencephalography (EEG) data collected in the time domain are run through a Fast Fourier Transform (FFT) to evaluate ranges of frequencies, or bands, including delta, theta, alpha, and beta bands. Alpha asymmetry scores are typically computed for frontal (F7, F3, F4 and F8) and temporal/pariental (T5, P3, P4, T6) EEG electrodes measured at the scalp. As alpha power is thought to reflect the inverse of cortical activity, right minus left hemisphere alpha power produces a metric suggestive of relatively greater left than right hemisphere cortical activity.

**Figure 2:**
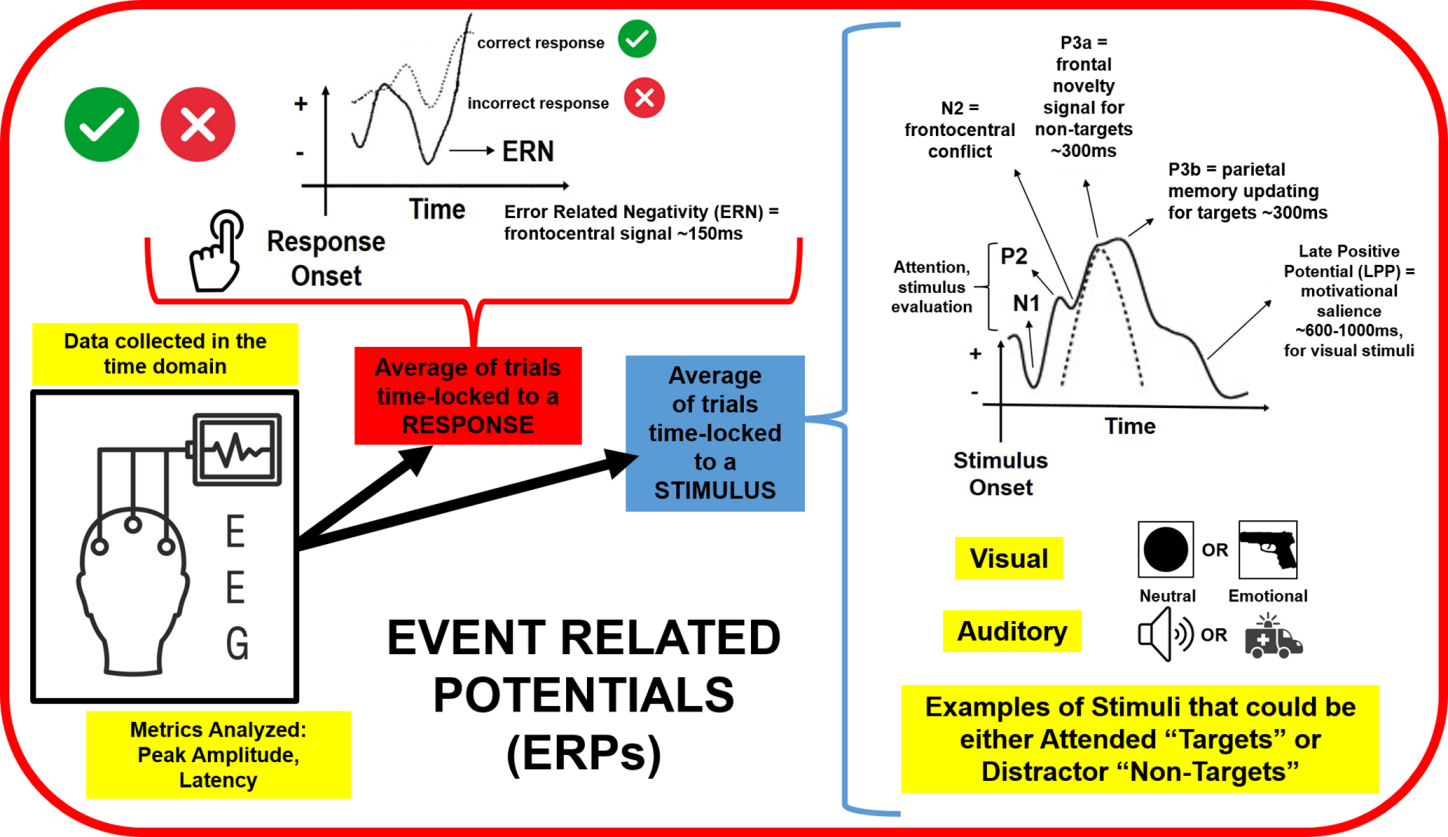
Continuous electroencephalography (EEG) data are segmented into trials time-locked to a particular stimulus or response, and then these trials are averaged together to evaluate the peak amplitude and latency of various event related potentials (ERPs). Auditory and visual stimuli that are neutral and emotional or trauma-related have been employed to study attention, stimulus evaluation, conflict and novelty processing, memory updating, and motivational salience as a function of trauma exposure. ERPs to incorrect responses have also been explored to evaluate links between trauma experiences and error monitoring.

EEG signals represent relative electrical potentials acquired over time at sensors placed on the scalp while an individual is in an uncontrolled resting state or an active task session. EEG signals can then be extracted for data analysis in the frequency domain, illustrated in **Figure 1**, and/or the time domain, illustrated in **Figure 2**. The spectral composition, or power spectrum, of the signal is then estimated using various fast Fourier transform algorithms. The most common metric employed in analysis is power within a particular frequency band. Differences in power between hemispheres are also quantified by calculating an asymmetry score metric. Additionally, peak frequency values within a band (identifying the frequency with the highest amplitude per individual) and connectivity (amplitude or power correlations between electrodes located in various scalp locations) can be quantified. EEG data are typically analyzed in the time domain by time-locking electrical signals elicited to a particular stimulus or response and then averaging these time-locked signals over multiple trials to produce an event related potential (ERP), which amplifies signals to an event while cancelling out random noise present on individual trials. We review trauma and PTSD literature for frequency and time domains separately below. On the whole, most EEG studies within this literature record data from low-density electrode montages, limiting spatial resolution of signals beyond anterior versus posterior, or frontal, central, temporal, and parietal versus occipital scalp locations.

## Electroencephalography Frequency Patterns Linked to Trauma Experience and Posttraumatic Stress Disorder Symptoms

**Figure 1** illustrates that continuous EEG recordings are typically examined as a function of particular frequency bands, such as delta, theta, alpha, beta, and gamma; frontal and parietal brain asymmetry is also computed by computing alpha band differences between the hemispheres. **Table 2** provides a summary of effect size magnitude for significant EEG frequency results, whereas **Table 3** provides detailed information for EEG studies examining trauma within the context of various frequencies, including those involving frequency bands outlined above [see also (139), for a review on resting state EEG frequency bands in psychiatric disorders]. On the whole, published reports linking EEG activity to PTSD diagnosis/symptoms are inconsistent, with the exception of alpha band-related parietal EEG asymmetry. The majority of EEG frequency studies collect brainwave recordings while individuals are in an uncontrolled “resting state” with their eyes closed or open, in the latter case focusing their eyes on a fixation cross in the center of a computer screen. Although it has been argued that these resting state recordings may reflect trait characteristics (140), research demonstrates that resting EEG consists of ~40–50% state variance, likely due to heterogeneity in individual participant cognitions (“mind wandering”) and arousal levels during recording (141). Inconsistent resting EEG results across studies may be due, in part, to state-related variations across individuals that dominate over clinical group or symptom differences.

### Delta Band

Whereas spontaneous delta band activity is associated with rapid eye movement sleep and may reflect processes implementing basic biological functioning (142), blunted delta oscillations elicited during various active tasks are thought to index deficits in cognitive functioning (143, 144). Delta oscillations Although greater early life trauma is linked to lower delta power (35), additional studies report no delta power differences between PTSD+ and PTSD− with or without trauma exposure (47, 53, 97). More research is warranted to determine whether early life trauma results in developmental disruptions to the brain that may be marked by heightened delta signals.

### Theta Band

Theta band oscillations are associated with cognitive processes such as working memory load and sequencing as well as encoding of spatial and temporal information (145–147). Similar to delta band findings, resting state theta band results are also mixed, with studies showing higher theta power in PTSD+ than PTSD− with and without trauma exposure (61, 97, 101), no differences between PTSD+ and PTSD− with or without trauma exposure (47, 53), or the opposite pattern, wherein greater early life trauma and PTSD+ status are both linked to lower theta power (35, 105). As PTSD+ were compared to PTSD− without similar trauma exposure in (105), perhaps trauma experience alone in some circumstances, but not PTSD, is linked to reductions in cognitive control processes indexed by theta power. Attenuated theta power may manifest in difficulty maintaining information in working memory or difficulty evaluating negative consequences of behavior, but more research on theta oscillations within the context of task-based paradigms may elucidate the role of theta signals in PTSD.

### Beta and Gamma Bands

Beta oscillations are thought to reflect active control of an individual’s current cognitive or motor state, as well as anticipation of changes to this state; it is argued that heightened beta power may reflect cognitive or behavioral inflexibility (148). Similar to delta and theta results, resting state beta band findings are incongruent, with two studies showing that early life trauma and PTSD+ status are negatively correlated with beta power (35, 98) but others reporting: 1) no differences between PTSD+ and PTSD− without trauma histories (53); or 2) opposite findings, with PTSD+ displaying greater beta power than PTSD− without similar trauma experiences (97). Faster than beta rhythms, gamma oscillations are present during various cognitive processes and are argued to reflect multisensory stimulus perception, evaluation, and representations in short- and long-term memory; similar to beta signals, gamma signals are also linked to processing of motor responses (149). One study reports that PTSD+ exhibit lower resting state beta and gamma band connectivity strength than PTSD− without trauma experiences, a neural pattern associated with greater depression, arousal, and other trauma symptoms (48). In contrast, another study reports that PTSD+ is linked to higher gamma band activity than PTSD− who had also experienced traumatic events (99). As of now, there is no cohesive theory explaining how alterations in these frequency bands relate to PTSD symptoms or trauma processing. Future evaluation of theta, beta, and gamma oscillations during specific cognitive or emotional tasks, as opposed to uncontrolled resting state recordings, may elucidate potential perceptual, motor, and memory impairments linked to PTSD.

### Alpha Band

Alpha is the most commonly studied of the frequency bands, with alpha power, peak frequency, asymmetry, and connectivity being investigated. It is argued that increased alpha power reflects suppression of task-irrelevant processes, whereas decreased alpha power reflects the release from inhibition, or in other words, degree of cortical activation (147, 150). For overall alpha power, null group findings between PTSD+ and PTSD− with or without trauma histories (47, 53, 97); again conflict with other studies demonstrating that early life stress and PTSD+ status are associated with lower alpha power (35, 46). Although PTSD+ status and early life trauma are both linked to higher alpha peak frequency (35, 47), it is unclear how peak frequency differences conceptually relate to trauma symptoms. In summary, the relevance of overall alpha power across the scalp to PTSD symptoms is unclear.

### Alpha Brain Asymmetry

Hemispheric differences in alpha band activity are often studied using an EEG asymmetry score, which subtracts left from right alpha power; as alpha power is thought to reflect the inverse of cortical activity, positive scores on this asymmetry metric indicate increased activity in the left hemisphere, and negative scores indicate increased activity in the right hemisphere (151, 152). Frontal EEG asymmetry patterns measured during a resting state are thought to reflect individual differences in motivational and emotional styles, with increased leftward activity indicating heightened approach-related motivation/emotion, and increased rightward activity reflecting heightened withdrawal-related motivation/emotion (140, 153). As core symptoms of PTSD and traumatic reactions reflect increased negative emotionality, it would be expected that presence of PTSD and/or severe trauma symptoms would be associated with relative right frontal asymmetry. Indeed, relative right frontal asymmetry is linked to greater symptom severity of: 1) childhood maltreatment in girls (40, 41) and 2) PTSD+ in a mixed-sex sample (44). Moreover, a novel longitudinal study measuring EEG asymmetry and clinical symptoms three times within a 2-year period indicates that for girls with low child trauma severity, rightward frontal EEG asymmetry across timepoints is associated with future development of PTSD (104).

However, results are far from consistent, particularly for males. Increased trauma severity in boys is associated with increased left frontal asymmetry (40); however, PTSD+ and PTSD− do not differ in males with a history of combat-related trauma (47). Mixed-gender samples also show conflicting findings, with PTSD+/PTSD− trauma-exposed individuals exhibiting greater left frontal asymmetry than PTSD− non-exposed participants (43), PTSD+ showing similar asymmetry patterns as PTSD− without traumatic histories (103), and non-significant relationships between frontal asymmetry and trauma, depression, and anxiety severity (100). Lack of consistent findings could be related to: 1) sex differences in frontal asymmetry previously reported in the literature, with depression linked to left frontal asymmetry in men but right frontal asymmetry in women (154), divergent patterns that may be obscured by not directly comparing males and females in statistical analyses; 2) use of a Cz reference montage (100), shown to be the least consistent in producing reliable asymmetry results (152); and 3) recruitment of “super controls” who differ on various personality/mood variables from PTSD+ individuals (103).

Some researchers argue that individual differences in motivation/emotion associated with EEG asymmetry may be more reliable and robust when measured during emotional challenges than resting states, as these challenges may directly engage frontal regions involved in these motivational tendencies (155, 156). Thus, with the context of active tasks, it would be expected that PTSD+ status and/or severity is associated with greater relative right frontal asymmetry, reflective of heightened withdrawal-related motivation/negative emotion processing. Indeed, studies demonstrate that higher right frontal asymmetry is linked to: 1) more severe PTSD, rumination, and depression symptoms when viewing negative images (43); 2) greater emotional intensity and physical reactions to trauma images (43); and 3) PTSD+ during trauma image viewing, compared to PTSD− (42).

Parietal EEG asymmetry, in contrast, is thought to index levels of arousal as opposed to valence, wherein greater right than left parietal activity reflects higher arousal (157, 158). Although depression is linked to under-arousal reflected by greater left than right parietal activity, findings are inconsistent and may be influenced by state factors such as recent caffeine intake (156). Although PTSD is characterized by increased negative affect (depression), hyperarousal (associated with anxiety) is also a core component of the disorder, suggesting that relative right parietal asymmetry may more accurately index PTSD symptoms. Three studies show results consistent with this hypothesis, wherein greater right parietal asymmetry is linked to greater childhood maltreatment severity in boys and girls (40), higher comorbid depression and arousal symptoms in PTSD+ women (45), and PTSD+ individuals, as opposed to PTSD− participants diagnosed with MDD who showed lower right parietal asymmetry (44). Moreover, recent work demonstrates that PTSD+ display greater midline-to-right hemisphere parietal alpha connectivity than PTSD− (101) as well as greater alpha suppression in right parietal regions (61), findings which may help to explain the right versus left hemisphere imbalance in the parietal lobes. In addition to resting state recordings, parietal asymmetry patterns emerge during emotional challenges, wherein PTSD+ display greater right than left parietal activity than PTSD− and higher PTSD symptom severity is linked to higher relative right parietal asymmetry during trauma image viewing (42). On the whole, state emotional challenges targeting approach and withdrawal mechanisms may provide more consistent, robust biomarkers of PTSD pathophysiology than EEG recorded during a resting state.

Finally, type of trauma experience may play a role in heterogeneity of EEG results. Although PTSD+ and PTSD− *combat veterans* do not differ in frontal or parietal alpha asymmetry (47), other studies indicate that PTSD− who have experienced other types of traumatic events (accidents and witness to injury/death) similar to PTSD+ exhibit greater left frontal alpha asymmetry than PTSD+, a pattern thought to reflect resilience, or protection against deleterious effects of stress (42, 43, 102). Perhaps prolonged exposure to certain types of trauma such as combat alters brain function regardless of the degree of clinical symptoms experienced, although more research is warranted to test this hypothesis.

### Unconventional Electroencephalography Analysis Approaches

#### Microstates

Traditional frequency band analysis consists of averaging power within a particular band over several minutes of EEG recording. It is argued that this trait-like measurement may miss out on faster, dynamic shifts in neural activity, termed microstates, which may capture variance linked to psychopathology. During a resting state, individuals transition to and from microstates, or specific patterns of electrical activity evident across the scalp, thought to reflect oscillations within specific networks (159). Microstates are quantified by calculating global field power, the standard deviation of electrical strength across all electrodes at a particular moment in time. Researchers can then plot global field power as a function of time across a set of frequencies (e.g., alpha band) to generate topographic maps that can be submitted to cluster analysis; results from this analysis provide information on how long each individual stays in a particular microstate and how often the person transitions in and out of these microstates (159). Much more research is needed to determine the functional relevance of each microstate and how it relates to various clinical constructs. A recent innovative study in this regard indicates that combat PTSD+ differ from combat-exposed PTSD− on three of eleven EEG microstates emerging from feature analysis; these microstates were then correlated with simultaneously recorded functional magnetic resonance imaging (fMRI) activity and PTSD symptoms to enhance interpretation of results (160). Specifically, two findings link EEG/fMRI activity with PTSD symptoms: 1) PTSD+ spend more time in a microstate associated with the medial prefrontal cortex (a component of the brain’s default mode network) than PTSD−, and within PTSD+, this greater duration is linked to higher PTSD symptom severity; and 2) PTSD+ spend less time in a microstate associated with insular and cingulate cortices (components of the brain’s salience network) than PTSD−, and within PTSD+ lower duration in this state is linked to higher anhedonia, or loss of pleasure (160). These results suggest that individuals with more severe PTSD symptoms are internally hyper-focused and paying less attention to externally relevant stimuli. If these microstates predict treatment outcome or can be changed as a function of treatment, EEG could be used to track these metrics.

#### Non-Linear Electroencephalography Dynamics

Examining relationships in brain activity between and within electrodes over time can highlight whether electrical complexity and connectivity between cortical regions are disrupted as a function of trauma. Compared to PTSD− without trauma exposure, civilian PTSD+ show higher connectivity (correlations between electrodes) within the left hemisphere but lower connectivity within the right hemisphere; this pattern is thought to reflect attenuated neural processing complexity in the right compared to the left hemisphere (161). Another study used non-linear modeling to determine that civilian PTSD+ exhibit lower complexity than PTSD− without trauma exposure in several electrode locations, most within the right hemisphere (162). These findings point to right hemisphere dysfunction in PTSD consistent with alpha band asymmetry results. A recent study also demonstrates that PTSD+ exhibit reduced functional connectivity within delta, theta, and beta bands than PTSD− without a trauma history; EEG source analysis localized one connectivity metric to a right centroparietal region, with lower connectivity linked to higher IES-R PTSD symptoms (163). These results point to the importance of investigating information processing impairments in brain networks as well as specific brain regions in the study of psychopathology more generally and PTSD symptoms in particular.

## Electroencephalography in the Time Domain: An Overview of Event Related Potentials

ERPs, illustrated in **Figure 2**, are segments of EEG that are averaged over repeated trials to characterize the neural response to a particular event, such as a stimulus, response, or absence of a response (e.g., inhibiting a button press). ERPs are typically analyzed by investigating differences in amplitude and latency between stimulus conditions and/or groups of subjects. ERP amplitude is often defined as the difference between a pre-stimulus baseline and the largest waveform peak within a particular time-frame, whereas ERP latency comprises the time from stimulus onset to peak amplitude (164, 165). The components we will review in relation to PTSD include N170, P200, N200, P300, N400, contingent negative variation (CNV), mismatch negativity (MMN), error-related negativity (ERN), and late positive potential (LPP). These particular ERP components are described within the context of relevant paradigms below. EEG recording montages vary to the degree that electrical signals at the scalp manifesting in ERPs are able to be localized to their sources within the brain. EEG/ERP source localization studies typically employ 64 or more scalp electrodes of spatial resolution to more accurately estimate the origins of electrical signals, often in conjunction with magnetic resonance imaging or magnetoencephalography methods. As EEG/ERP source localization studies specifically within PTSD samples are relatively uncommon, for various ERP components we cite their potential sources based on other healthy or clinical samples.

## Event Related Potential Markers of Trauma Experience and Posttraumatic Stress Disorder Symptoms

Common paradigms employed to evaluate ERPs as a function of trauma and PTSD status include auditory and visual oddball, Stroop, flanker, go/nogo, facial identification, and passive listening/viewing tasks. Historically, oddball tasks have focused on basic tones or visual shapes not associated with trauma or emotional significance; these tasks help to address whether a particular processing deficit generalizes across all stimuli, or is just specific to trauma. While **Table 2** provides an overview of effect sizes for auditory and visual ERPs in the same chart, going forward we review ERP component findings for auditory (**Table 4**) and visual (**Table 5**) modalities separately. Below we cluster findings as a function of stimulus modality and the specific function(s) a particular ERP component is thought to capture (attention, working memory, inhibition, emotion regulation, or reward processing). Paradigms employed by ERP researchers vary in complexity depending on the question of interest. This could include use of passive viewing paradigms versus those involving action selection and/or inhibition of these actions (i.e., Oddball tasks). Because of the differences in neural resources required for the latter process, we include inhibition as a separate section from attention and working memory paradigms. Within each type of EEG paradigm, researchers often use either neutral or trauma-relevant stimuli to ascertain the specificity versus generalized quality of findings. Results using neutral or trauma-relevant stimuli are discussed within the sections describing each type of paradigm (i.e., attention and working memory, inhibition, emotion regulation).

### Auditory Attention and Working Memory

During passive auditory tasks, participants listen to tones of various intensities and are not required to mentally count stimuli or make a behavioral response; within these tasks, the brain’s selective attention to stimulus characteristics is the primary focus. P200, or P2, is a positive component peaking from 150 to 275 ms, thought to be associated with selective attention, detection, and retrieval in short-term memory evident throughout frontocentral electrodes (166, 167). N200, or N2, occurs closely following P2 and is a negative component occurring around 200 ms in prefrontal, anterior cingulate, and superior temporal cortices (168). N2 detects deviant stimuli while attention is fixed upon a standard stimulus (164, 169). One study indicates that PTSD+ exhibit lower P2 amplitude to high intensity (deviant) tones than PTSD− without a trauma history, a pattern linked to lower depression and PTSD symptom severity (51). In contrast, a second study with a larger sample demonstrates the opposite effect, wherein PTSD+ display greater P2 and N2 amplitudes to higher intensity tones than PTSD− who do have a trauma history, a pattern associated with heightened re-experiencing symptoms within PTSD+ (113). These two studies differ in type of trauma (combat versus child physical and sexual abuse) as well as inclusion criteria for their comparison groups, factors that may contribute to divergent findings.

Two-stimulus auditory oddball tasks require participants to press a button or mentally count infrequently occurring target tones in-between more frequent standard tones (thereby paying more attention to target than standard stimuli). The MMN ERP component captures the difference in neural processing between these standard and target stimuli. MMN is usually largest in amplitude over the central and frontal electrodes at 120 ms, suggesting that it is the mismatch detector, a pre-attentive processing of deviance in short-term memory. MMN is also thought to be reflective of a sensory memory system (62, 109, 114). With respect to early sensory processing of deviant stimuli, PTSD+ exhibit greater MMN amplitude to targets than standards when compared to PTSD− with or without trauma histories (61, 62, 109, 114); furthermore, exaggerated MMN amplitude is linked to greater PTSD symptom severity (62). In contrast to MMN results, N2 amplitude results also indexing deviance are mixed, with PTSD+ exhibiting N2 target amplitudes higher (50, 62) and lower (53) than PTSD− without trauma histories, paired with longer N2 latency (50, 108). As greater trauma severity is linked to lower N2 target amplitude (35), it would be worth investigating N2 amplitude further in studies explicitly matching PTSD+ and PTSD− on trauma history.

In addition to MMN, P2, and N2 ERP components, P300, or P3, is a positive waveform peaking around 300 ms, is elicited to in response to an attended target stimulus, prior to response generation. P3 is thought to reflect memory updating, information delivery, stimulus evaluation, active discrimination, and motivational salience on the basis of stimulus context (164, 165, 169). P3 is divided into two subdivisions: P3a and P3b. P3a is typically largest in frontal and central areas to non-target stimuli in more complex oddball paradigms (it is not included in the two-stimulus oddball), whereas P3b is activated in the temporal, parietal, and cingulate cortices and is typically largest to oddball targets when compared to standards as well as other stimuli (168, 170). The most robust outcome of two-stimulus oddball studies is that PTSD+ exhibit lower target P3b amplitude than PTSD− with trauma histories (54, 55) and PTSD− without trauma histories (50, 53, 56, 58, 108) with lower P3b amplitude linked to heightened avoidance/numbing, re-experiencing, and hyperarousal symptoms (50, 54, 58). Of note, acute stress disorder (ASD), thought to be a precursor to PTSD, is linked to greater target tone P3b amplitude than PTSD+ and PTSD−, and although the latter groups do not differ, direction of group means suggests that PTSD+ displays the lowest P3b amplitude out of the three groups (107). These results point to the fact that trauma recency may be an important moderator of the brain’s auditory attention response. In contrast, all but one study (50) found no differences in P3b target latency as a function of PTSD diagnosis (51, 54, 56, 58). In summary, most findings point to reduced neural resources devoted to auditory working memory updating, reductions that are worsened as a function of symptom severity, specifically for intrusions, dissociation, and hypervigilance.

In three-stimulus oddball tasks, in addition to infrequent target and frequent standard stimuli, an infrequent non-target is added that, unlike the target, does not require a behavioral response. Addition of this non-target allows researchers to study degree of neural resources devoted to relevant (target) versus irrelevant (non-target) attentional capture while simultaneously controlling for frequency of stimulus presentation. Four-stimulus oddball tasks include two non-targets instead of one, often comparing stimuli with differing emotional qualities (positive versus negative emotion). On the whole, the addition of non-target stimuli results in less consistent results as a function of PTSD status, although these studies are predominantly characterized by combat trauma samples being compared to PTSD− with trauma histories. Although some work indicates that P3b target and P3a non-target amplitudes do not differ between PTSD+ and PTSD− twins with and without trauma histories (110) or PTSD− with trauma experience (111, 115), additional studies report that PTSD+ exhibit lower or higher (49) P3b target amplitude than PTSD− who have experienced trauma. However, a small sample of PTSD+ shows auditory P3a reductions as a function of eye movement desensitization and reprocessing (EMDR) therapy, whereas a PTSD− administered sham therapy showed similar P3a amplitudes pre- and post-assessments (112). Perhaps type of trauma plays a role in inconsistent findings, as Metzger et al. (57) include childhood sexual abuse as well as combat trauma, whereas Metzger et al. (49) focus solely on combat-related PTSD.

It is important to note that non-target results discussed thus far consist of deviant tones unrelated to traumatic events; perhaps trauma-relevant non-target stimuli evoke a more powerful neural response than non-relevant tones. To this end, higher P3a trauma-related non-target amplitudes are associated with greater hyperarousal symptoms in individuals located near (59) but not far from a natural disaster (60). Furthermore, greater PTSD avoidance and hyperarousal symptoms related to a traumatic fire are linked to greater P2 and P3a amplitudes to both positively and negatively valenced non-target stimuli (106), suggesting that exaggerated attention is paid to emotionally arousing stimuli in PTSD, particularly for individuals experiencing hypervigilance to threat.

### Auditory Inhibition

Within the context of an inhibition paradigm, two mismatched tones (of different frequencies) signal that the participant should make a behavioral response (go), whereas two matched tones (of the same frequency) signal the participant to withhold a behavioral response (nogo). High levels of comorbid depression and trauma in a non-clinical sample are associated with longer frontal but shorter centroparietal P3 latencies to go stimuli, but it is unclear whether these findings are driven by trauma, depression, or both (63). A more complex auditory go/nogo variant consists of a continuous performance task wherein participants hear a stream of letters and must press a button (go) when they hear an “X” when it occurs after an “A,” inhibit a button press when they hear any other letter following an “A” (nogo), and ignore task-irrelevant letters (non-targets). Despite no group differences in P2 or P3 amplitude to go or nogo stimuli, PTSD+ exhibit longer P3 latency to nogo stimuli than PTSD− without trauma history, a pattern that is linked to greater hyperarousal symptoms. Moreover, PTSD+ show greater frontal P3a amplitude to irrelevant non-targets than PTSD− (91). These findings, while limited, suggest that trauma is associated with delayed stimulus discrimination and greater resource allocation to novel distractors.

### Visual Attention and Working Memory

Substantially more ERP trauma research has focused on attention and working memory processes within the visual modality than the auditory modality, with the majority of these studies measuring brain responses to emotionally valenced images or faces. Visual stimuli elicit P2, N2, and P3 amplitudes similarly to auditory stimuli, although the latency of component onset may be slightly delayed compared to auditory ERPs. In addition to the aforementioned components elicited by auditory stimuli, the LPP, a late positive ERP component visible from 400 to 1,000 ms, is modulated largely by the emotional content of visual stimuli; positive or negative images elicit larger LPP amplitudes (greater positivity) than neutral images (171). Simultaneous EEG and fMRI recordings demonstrate that LPP amplitude is positively correlated with signal increases in visual cortex, amygdala, and prefrontal cortex (172). With respect to these ERP components, studies involving passive image viewing provide mixed results. Although greater PTSD avoidance symptoms are associated with lower P3 and LPP amplitudes for traumatic than neutral images (75), PTSD+ show no differences from PTSD− in LPP amplitude to positive, trauma, or neutral images, despite exhibiting attenuated P2 amplitudes to these stimuli (67). A novel study was able to study the same individuals before and after their experience with a natural disaster, demonstrating that although PTSD− exhibit smaller LPP amplitude to negatively valenced images after as opposed to before the trauma, PTSD+ display no change in LPP amplitudes pre- to post-trauma, signaling exaggerated responses to aversive events (119). It may be the case that PTSD characterized by exaggerated avoidance of thoughts and feelings reminiscent of trauma reduces neural resource allocation to threatening stimuli, at least in the short term.

Research suggests that face processing may be altered as a function of exposure to trauma, although findings are inconsistent as to whether alterations are present across faces or specific to emotional expressions signaling threat. N170 is a visually evoked negative component occurring at 170 ms that is enhanced to face stimuli (173). Conflicting N170 results exist as a function of traumatic experiences. Although childhood trauma is linked to smaller N170 amplitude to angry/fearful as opposed to happy faces (83, 85), both combat-related trauma and PTSD+ are associated with heightened N170 amplitudes to faces more generally (84) but no N170 amplitude change to angry faces as a function of pre- versus post-deployment in PTSD+ and PTSD− with similar combat trauma (65). The P2 literature is equally messy in that PTSD+ display either attenuated P2 amplitude to happy faces (66, 121) but enhanced P2 amplitude to various faces when compared to PTSD− with traumatic brain injury (TBI) (123). Finally, PTSD+ display greater P2 amplitude to angry faces than PTSD− post- as opposed to pre-deployment (65). With respect to P3 differences, PTSD+ display longer frontocentral P3 latency to happy faces than PTSD− with history of trauma (99) as well as larger P3 amplitude to neutral than angry faces during a dot probe task (71). During later elaborative processing, PTSD symptoms in combat veterans are linked to smaller LPP amplitude to angry faces (66, 78, 79). Inconsistent results may be due, in part, to varying task requirements, type of trauma studied, and exclusion criteria related to medication status, SUD, and depressive symptoms.

Additional visual discrimination paradigms pair emotional, neutral, and/or trauma images with an active task requirement that involves making a choice based on other irrelevant features of the images (e.g., whether an image includes food, contains a certain color, or is located in a specific spatial location). Trauma experience more generally is associated with larger P2, P3, and LPP amplitudes to trauma as opposed to neutral images (64, 127) as well as larger LPP amplitude to negative than neutral images (80, 174); the latter pattern also predicts higher future externalizing symptoms in those reporting the most severe trauma symptoms (80). Clinical studies, on the other hand, provide inconsistent results, with PTSD+ showing no difference in P3 amplitude to emotional or neutral images than PTSD− without a history of trauma, but displaying heightened LPP to positive images (77) or no group differences in LPP amplitude to threat (117). In contrast, PTSD+ display greater P3 and LPP amplitudes to food, neutral, and trauma images than PTSD− who share a history of combat-related trauma (122); as both studies possessed small sample sizes, it is unclear how to reconcile these results.

An ERP component employed to study anticipation of an upcoming stimulus is the CNV, a slow negative wave associated with working memory and motor/perceptual timing (175). While typically recorded in frontocentral regions, multiple brain areas are thought to contribute to CNV formation including prefrontal, primary motor, and primary somatosensory areas (176). During a choice reaction time task, PTSD+ show greater CNV amplitude to negative stimuli than PTSD− without a history of trauma, with higher PTSD severity and re-experiencing symptoms linked to higher CNV amplitude (82). These findings suggest that exaggerated neural resources are being allocated to past and future aversive events as a function of PTSD, although additional research is warranted to determine if results generalize to trauma experience or solely a function of PTSD.

Two, three, and four visual oddball conditions are analogous to auditory oddball designs, with most visual studies focusing on P3 differences as a function of trauma or PTSD diagnosis. Two-stimulus oddball studies report that lower target P3b amplitude differentiates PTSD+ from PTSD− (70) and also predicts future conversion from PTSD− to PTSD+ (74). In contrast, for individuals who have experienced combat trauma, P3b amplitude increases over time parallel reductions in PTSD and depression symptoms within the same window, whereas no changes in P3b amplitude are associated with increased symptoms (76). Three- and four-stimulus oddball paradigms demonstrate that PTSD+ exhibit larger P3a amplitudes to traumatic non-targets than PTSD− both with and without trauma histories (68, 72, 124). Moreover, both PTSD+ and trauma history appear to be associated with smaller P3b target amplitude (68, 73). Findings for P3 latency are mixed, with studies reporting slower (124) or faster P3a latency to trauma (72), paired with slower (116) or faster (68) P3b latency to targets.

In addition to oddball tasks, researchers have used various word-based paradigms to assess short-term memory function linked to trauma experience. When performing a Sternberg item-recognition task involving the maintenance of a word list in short-term memory and then differentiating these words from new words, PTSD+ with comorbid TBI and PTSD− with trauma do not differ in P3 amplitude to old versus new words when they perform this task in isolation; however, completing this task in the presence of additional cognitive load, P3 amplitude fails to differentiate old from new words in PTSD+ with TBI but not PTSD− with trauma (118). It is difficult to attribute study results specifically to a PTSD diagnosis, given that TBI presence differed between groups. During a 1-back working memory task involving letters, unmedicated PTSD+ exhibited larger P3 target amplitude than medicated PTSD+ despite no differences from PTSD− (69). During a declarative memory paradigm, PTSD+ display larger P3 and LPP amplitudes to trauma-related questions than PTSD− without trauma, but PTSD− with trauma did not differ from either group; in contrast, PTSD+ and PTSD− with trauma show smaller frontal CNV amplitude to trauma questions than PTSD− without trauma (125). On the whole, results do not suggest a basic visual working memory deficit specific to PTSD.

Within individuals who experienced a natural disaster, P2 and P3 amplitudes are larger for trauma-related than trauma-unrelated words, signals localizing to parahippocampal gyrus (126). N400, or N4, is a negative ERP deflection that is largest in response to unexpected information (often related to language processing) that is localized to middle, superior, and inferior temporal regions as well as dorsolateral prefrontal cortex and thought to relate to semantic memory retrieval (177). PTSD+ comprised of various traumas display smaller N4 amplitude to threatening sentence endings during a sentence completion task than PTSD−, but no differences for expected or unexpected sentence endings (120), suggesting that trauma-related information is more readily accessible in semantic memory, and is therefore less unexpected. Furthermore, a recent study demonstrates that within trauma survivors, larger N400 amplitudes to negative sentence endings is linked to greater negative cognitions about the world as a result of trauma (86).

### Visual Inhibition

In the color-word Stroop paradigm, individuals are instructed to read color words (congruent trials) or name the color in which these words are written (incongruent trials), and the Stroop effect involves subtracting reaction time of congruent from incongruent trials to obtain a measure of response interference (178). For example, if “red” is written in green ink, congruent trials require the participant to select “red” as the correct response, whereas for incongruent trials, the correct answer would be “green.” In a modified emotion-word Stroop paradigm, color-related words are replaced by positive, negative, neutral, and/or trauma-related words, and individuals are required to select the correct color in which each word is written; longer responses to emotional than neutral words are thought to reflect greater attention paid to emotional words. Color-word Stroop results indicate that greater trauma symptoms are associated with lower right prefrontal negativity from 400 to 600 ms for incongruent as opposed to congruent stimuli (133); these results point to impaired inhibitory processing. Three emotion-word Stroop studies also indicate that PTSD+ display smaller P3 amplitude than PTSD− with and without trauma histories across trauma, emotional, and neutral conditions (57, 87, 128), although an additional study finds that individuals with a trauma history exhibit greater P3 amplitude to positive than negative words when compared to subjects without trauma, findings localized to parahippocampal gyrus and cuneus brain regions (88). On the whole, findings suggest that PTSD is characterized by reduced resources devoted to suppression of conflicting information within the context of this paradigm.

Visual go/nogo paradigms are similar to those within the auditory modality, wherein one or more cues are linked to an active behavioral response (go), and another cue or cues are linked to withholding of a response (nogo). Some variations of this paradigm also include distractor stimuli to examine whether participants are diverting neural resources to task-irrelevant information. The stop signal task also requires behavioral inhibition on some trials, but the design is slightly different: participants press a right button to one visual cue and a left button to another visual cue (go), and on a certain percentage of trials, they hear an auditory tone (signal) requiring them to withhold their response (stop). These paradigms tend to elicit larger frontal N2 amplitude to nogo/stop trials than go trials (179), which within this context is thought to reflect a heightened response to conflict (177). In addition, larger frontocentral P3 amplitude to nogo/stop trials than go trials is thought to more directly reflect inhibition of an overt motor response (181). Although some research indicates that PTSD+ do not differ in N2 or P3 amplitude to go or nogo stimuli from PTSD− with trauma histories (89, 91), within the context of a combat sample with comorbid traumatic brain injury (TBI), PTSD+ display larger N2 amplitude, localized to anterior cingulate cortex, than PTSD−, a pattern that is also positively correlated with PTSD symptom severity (134). However, heightened frontal P3 amplitude to nogo trials is linked to greater PTSD symptoms within a sample of police officers (90), and PTSD+ with combat trauma display greater frontal P3 amplitude to distractors than PTSD− without trauma (91). Moreover, studies indicate that delayed P3 nogo latency is associated with greater PTSD hyperarousal and avoidance (91, 92), whereas delayed distractor latency is linked to greater PTSD re-experiencing (91). One additional ERP component that is often computed for inhibition-related tasks is the ERN, a negative deflection occurring 150 ms after commission of an error, which is localized to prefrontal and anterior cingulate cortices (182). Recent work demonstrates that children with higher stress paired with larger ERN amplitude during a go/nogo task show greater internalizing symptoms following a natural disaster (94), although it is unclear whether this finding relates to trauma more generally as opposed to PTSD in particular.

The Eriksen flanker task involves suppressing distractor stimuli that are either the same (congruent) or different (incongruent) from a central target stimulus; typically participants press a button indicating the direction of the target (left or right) and this task produces more errors for incongruent than congruent trials, due to the added difficulty of inhibiting conflicting information to achieve accurate task performance. Three studies indicate that combat-related PTSD+ do not differ in ERN amplitude during flanker performance than PTSD− either with or without trauma history (129, 132, 135). In contrast, within a non-clinical sample of individuals who experienced various types of trauma, larger ERN amplitude was positively correlated with PTSD hyperarousal symptoms, but not overall symptom severity (131); taken with ERN findings for the visual go/nogo task, perhaps heightened ERN reflects exposure to trauma more generally, although this hypothesis warrants further testing. Consistent with this idea, a recent investigation reports that greater combat exposure is associated with larger ERN amplitude after accounting for variance linked to anxiety and PTSD symptoms (93). Recent work suggests that the ERN amplitude itself may not be larger as a function of degree of childhood trauma, but instead the response to correct trials, the correct related negativity (CRN) amplitude, may parallel a greater degree of stressful life events (130). As CRN is sometimes employed in the difference score to calculate the size of the ERN, analysis of individual CRN and ERN waves may clarify patterns of results. Within a flanker task incorporating emotional distractors, PTSD is associated with heightened threat-related frontocentral positivity evident several hundred milliseconds post-stimulus (95).

Inhibition deficits as a function of a PTSD diagnosis appear to be somewhat paradigm- and ERP-component specific, with the largest effects for: a) a modified version of the Stroop task involving emotional, trauma, and neutral words; and b) P3 amplitude to words or distracting stimuli. Findings from other inhibition-relevant tasks do not point to a deficit in neural resources dedicated to monitoring for errors or withholding a behavioral response.

### Emotion Regulation and Reward Processing

As few ERP studies have examined the impact of trauma or PTSD on emotion regulation and reward processing, studies utilizing these types of paradigms are reviewed together. During a directive emotion regulation paradigm, individuals with combat trauma view negative pictures within two contexts: 1) where they maintain negative feelings elicited by the pictures; and 2) where they use cognitive reappraisal strategies to lessen the emotional impact of the images. Although PTSD+ and PTSD− do not differ in LPP amplitude to negative pictures before or during the reappraisal condition, PTSD+ display lower LPP increases than PTSD− for the negative-maintain condition (96). However, within a larger group of trauma-exposed combat veterans, less change in LPP amplitude during reappraisal over time parallels greater increases in PTSD avoidance, re-experiencing, and hyperarousal symptoms (136). These findings suggest that resources dedicated to adaptive emotion regulation are not impaired as a function of PTSD diagnosis, but that less LPP change over time results in increased PTSD symptoms as a function of traumatic exposure. During a delay discounting paradigm, trauma survivors display larger P3 amplitude to rewards and losses than individuals without trauma histories (137), but it is unclear as of yet whether this pattern is specific to PTSD. Finally, during reinforcement learning, ERP source localization analyses indicate that individuals with comorbid trauma and past MDD display greater anterior cingulate cortex signals to novel stimuli than individuals who meet criteria for past MDD+ but do not have a trauma history (138); again, it is unclear whether this finding globally relates to trauma history as opposed to PTSD.

## Synopsis and Clinical Implications

From a categorical approach to psychopathology, the majority of EEG/ERP studies do not produce clear-cut diagnostic markers of PTSD. On the whole, the electrophysiology literature primarily reflects inconsistent findings when comparing PTSD+ and PTSD− individuals; this lack of consensus is not that surprising, given that 70,000+ symptom presentations that can result in a DSM-IV PTSD diagnosis, and over 600,000+ symptom configurations can manifest in a DSM-5 diagnosis (183). Samples across studies likely differ on the severity of depression, avoidance, hyperarousal, re-experiencing, and other comorbid symptoms that could impact results. With respect to additional clinical issues, **Tables 3****–****5** illustrate that the majority of studies investigating trauma exclude individuals with medical and neurological problems (including brain damage) and at least some degree of psychiatric comorbidity (often psychotic and substance use disorders) from participation, reducing some influence of potential confounds; however, these exclusions can also limit the generalizability of findings, particularly given that PTSD is often comorbid with other mood, anxiety, and substance use disorders.

Discordant findings may also be related to small sample sizes, medication effects, time since trauma (e.g., early life stress being recalled in adulthood versus assault reported last year), type of trauma experienced (e.g., accidents versus assault or combat), and potential sex differences. Approximately one-third of the studies we reviewed explicitly address medication effects in their inclusion/exclusion criteria. Furthermore, only 23% report trauma recency; of those that do, substantial heterogeneity exists for the time since trauma exposure, ranging from 6 months to 10 years. In addition, only 10% of studies include biological sex in statistical analyses, despite the fact that EEG studies find more robust relationships between psychopathology and brain activity in women than men [e.g., Refs. (154, 184)]. Only 45% of studies compared PTSD+ to PTSD− who had actually experienced a similar traumatic event, thereby better isolating the impact of exaggerated responses to the trauma (clinical symptoms) as opposed to the trauma experience itself. Given considerable heterogeneity across individuals diagnosed with PTSD, it may be more realistic to focus on patterns of brain activity that are linked to specific symptoms of distress/impairment to be targeted in biological and psychological interventions. With large enough samples, medication presence/absence, sex, and time since trauma can all be included within models investigating relationships between brain activity and symptom dimensions.

Furthermore, although we attempted to illustrate the magnitude of various EEG and ERP relationships to trauma and PTSD in **Table 2**, results from **Tables 3****–****5** demonstrate that many of these studies do not report exact *p*-values and/or effect sizes, limiting quantitative conclusions regarding which electrical patterns are the most robust predictors of PTSD symptoms and/or outcomes. The majority of the effect sizes included in **Tables 3****–****5** were calculated by the senior author based on means/standard deviations or correlation coefficients included in each published article; in other words, these studies are not explicitly reporting effect sizes in their articles. To propel the field forward and encourage replication and ease of comparison, it is crucial that studies report effect sizes so that researchers and clinicians can gauge magnitude of various findings.

When we take categorical and dimensional EEG/ERP approaches together, four main patterns emerge. First, rightward frontal asymmetry has been identified as relating to PTSD diagnosis and symptoms and more specifically, social isolation/withdrawal and heightened negative affect. Given that these dimensions span not only PTSD but also other disorders such as MDD and PD suggests this may serve as a transdiagnostic marker of symptom severity. Future research can evaluate whether PTSD treatment interventions reduce this asymmetry as well as withdrawal-related symptoms of depression, rumination, and self-blame.

*Second, PTSD diagnosis, symptoms, and trauma severity are linked to rightward parietal brain asymmetry.* Research indicates that higher alpha power in right parietal cortex reflects greater internal attentional focus and orienting away from external distractors (185). As PTSD symptoms are associated with relatively lower alpha power (greater cortical activity) in right than left parietal regions, this imbalance may be linked to difficulty orienting attention away from distracting stimuli in the environment that are irrelevant to goal-directed behaviors. As individuals with PTSD have difficulty differentiating between safe and threatening cues, overestimate the probability of threatening events, and generalize threat responses across similar types of stimuli (186), it is possible that right parietal dysfunction contributes to or reflects this generalized hypervigilance and threat overattribution. A recent fMRI study demonstrates that within a sample of combat veterans, greater pre-treatment left inferior parietal activation during a stop signal task is associated with greater PTSD symptom reductions as a function of psychotherapy (187). Taken together, studies point to hemispheric patterns of parietal asymmetry as promising diagnostic and predictive markers of PTSD symptoms and treatment outcome, although more research is needed to replicate and extend these findings.

Third, individuals with PTSD show attenuated allocation of neural resources (often within parietal cortex) to process auditory/visual target (i.e., goal-relevant) and trauma stimuli within the context of memory updating (P3b amplitude) and motivational attribution (LPP amplitude; see **Table 2** for 20+ effects using at least one of these metrics); this effect is especially pronounced for those endorsing high avoidance symptoms. Perhaps exposure therapy could be enhanced by augmentations that bolster neural circuitry to enhance working memory and motivational focus (188).

Fourth, exaggerated processing of novel, emotional, trauma, and/or task-irrelevant auditory and visual stimuli specifically within frontocentral brain regions (CNV, MMN, and P3a amplitudes) is linked to greater PTSD re-experiencing, hypervigilance to threat, and overall symptom severity. These findings point to PTSD as a disorder of attention dysregulation—difficulty differentiating between task relevant and irrelevant information—that could contribute to problems concentrating and focusing on daily tasks (family/social interactions, school, or work) instead of distractors that could be interpreted as threatening and trauma-relevant (189). These distractors may trigger further intrusions and negative emotions that individuals find difficult to control.

## Reducing Impact of the Electrical Aftermath

Frontoparietal circuitry important for attentional processing across various tasks appears to be disrupted as a function of PTSD symptoms. Do changes in this circuitry parallel symptom reductions and can ERPs predict treatment response? Recent work demonstrates that the answer is yes on both counts. For instance, MMN and P3a amplitudes are malleable as a function of auditory-based targeted cognitive training in individuals with schizophrenia, with ERP changes predicting verbal learning improvements and reductions in clinical symptoms (190, 191). Moreover, P3b amplitudes predict treatment outcome and relapse for individuals with SUDs (192). Within trauma samples, fMRI research indicates that psychological and biological interventions appear to modulate activity and processing in frontal and/or parietal regions, resulting in promising PTSD symptom reduction [e.g., Ref. (2)]. Limited EEG research on prediction of treatment outcome suggests that: 1) CBT may result in a reduction of right frontal activation to trauma-relevant stimuli [an effect correlating with symptom reduction (193)]; 2) eye movement desensitization and reprocessing (EMDR) may elicit a reduction of the P3a component, suggesting a normalization during orienting to novel stimuli (112); and 3) transcendental meditation may increase EEG power within low-frequency bands (194). However, these studies suffered from small samples (less than 20 per group) and/or lack of comparison groups; thus, further work is needed in larger studies to assess the robustness of these findings. In the sections below we explain potential novel treatments that may target the EEG components implicated in the PTSD literature and highlight how EEG/ERP methodology can play a role in alleviating aversive symptoms linked to trauma exposure.

### Brain Stimulation

Noninvasive brain stimulation techniques such as repetitive transcranial magnetic stimulation (rTMS) and transcranial direct current stimulation (tDCS) show promise for altering attentional deficits linked to prefrontal cortex dysfunction. rTMS delivers a fluctuating magnetic pulse typically within the range of delta, theta, and alpha frequency ranges (1–10 Hz) to produce changes in neural activity; particular cortical regions are often targeted *via* the scalp with the use of an EEG cap. Although traditional rTMS requires 20–30 min to produce effects, theta burst stimulation (TBS) is a type of rTMS that applies continuous 5 Hz pulses to the brain, produces neural changes under 5 min, and is shown to influence performance on executive function tasks, particularly those involving working memory (195). Although no published studies have reported TBS results within samples of PTSD patients, a recent meta-analysis (196) indicates that frontal TBS effectively decreases depression symptoms that are often elevated in individuals with PTSD. Whereas rTMS/TBS applies magnetic pulses to the cortex, tDCS applies low frequency electrical currents *via* electrodes to a particular brain area to induce intracerebral current flow (197). Within combat veterans with PTSD, brain stimulation of prefrontal cortex alone or paired with cognitive therapy is associated with P3a normalization (reductions) to trauma-related stimuli [tDCS: Ref. (198); rTMS: Ref. (199)] as well as greater PTSD symptom reductions than cognitive therapy plus sham rTMS (200); available work suggests that targeted stimulation of right prefrontal regions may provide a more robust effect than left or bilateral stimulation (199, 200).

### Real-Time Electroencephalography Neurofeedback

Another promising technique for reducing PTSD symptoms is real-time EEG neurofeedback, wherein patients are given instructions to increase or decrease activity within a certain frequency band at a particular cortical region; EEG data are processed immediately during recording and patients are notified of their degree of success in changing their activity. In some contexts, patients are provided with examples of cognitive/emotional strategies that may assist in changing their brain activity, such as thinking of positive memories or future plans, or engaging in a working memory task. There have been recent reviews summarizing findings from clinical outcome studies using EEG neurofeedback for PTSD (201, 202). These reviews highlight the limitations of the field, including small sample sizes, lack of placebo controls, and/or lack of randomization to intervention conditions. The protocols often include training related to alpha/theta wave ratio (aiming to upregulate theta and downregulate alpha), alpha waves only, or thalamic inhibitory mechanisms. Initial evidence suggests that EEG biofeedback may result in PTSD symptom improvement, but findings are far from consistent across studies.

The use of fMRI concurrently with EEG may enhance the spatial targeting of neurofeedback. One research team recorded fMRI before and after 30 min of EEG neurofeedback focused on suppression of parietal alpha band activity in individuals with PTSD; within this study, patients were not informed of particular strategies to use or which frequency band to change, but were just presented with visual feedback of a spaceship that moved when participants were in the desired zone of brain activity (203, 204). This EEG neurofeedback targeting alpha band activity was associated with lowered hyperarousal symptoms, as well as enhanced connectivity between the prefrontal cortex and the amygdala, a subcortical brain region implicated in fear processing and aversive memories that is often overactive in PTSD (203, 204). As higher coactivation of prefrontal cortex and amygdala is linked to lower negative affect when healthy individuals are asked to regulate their emotions to negative stimuli (205), enhancement of this connectivity in PTSD patients may also assist in alleviating their symptoms of negative affect. As frontoparietal attentional mechanisms, particularly within the alpha band, are disrupted in PTSD patients and linked to hyperarousal, negative affect, and avoidance, available research suggests that EEG alpha neurofeedback may provide a “reset” for these mechanisms and reduce these aversive symptoms. For instance, neurofeedback using EEG/fMRI is associated with reduced PTSD symptom severity and greater leftward and bilateral prefrontal functional connectivity (203, 206). With respect to parietal regions, right temporal-parietal EEG neurofeedback is linked to lower tension, emotion dysregulation, and affect instability in PTSD patients than a wait-list control condition (207).

In sum, although neurofeedback is associated with PTSD symptom reduction across multiple studies, additional randomized control trials are warranted to determine how well neurofeedback performs in comparison to other treatments (201). While combined EEG and fMRI recordings allow for more precise spatial localization of brain changes than EEG alone, fMRI methodology is much more expensive and less mobile than current EEG systems, many of which are portable and can be easily set up for use in a clinic, university, or hospital. As EEG-fMRI evidence accumulates regarding brain mechanisms/circuitry involved in neurofeedback and symptom changes, we forecast that EEG neurofeedback may be more commonly used without fMRI recording to augment psychotherapy and/or pharmacotherapy treatment for PTSD.

### Developing Precision Medicine Approaches to Treatment: Clinician Recommendations

How can our findings inform clinicians working with patients who have experienced significant trauma? Just as there are many pathways to a PTSD diagnosis post-trauma, there is no “one size fits all” treatment for PTSD. Just as neuropsychological tests can be used by clinicians to estimate the degree and type of executive functioning deficits for an individual with PTSD (187), we posit that an assessment of EEG frequency and auditory/visual ERP metrics of attention/working memory could provide important insights regarding impairments that could be targeted in personalized interventions. Notably, the current literature is not at the point in which findings can be used to directly inform treatment decisions or approaches with individual patients. However, there are several potential avenues in which EEG/ERP studies could soon have clinical relevance and thus, could be of top priority for future clinical research.

Our review of ERP studies suggest that some individuals with PTSD, particularly those experiencing hyperarousal, intrusions, and dissociation, show specific deficits in auditory working memory that could potentially be targeted by cognitive training similar to that employed by researchers to reduce auditory impairments in patients diagnosed with schizophrenia [e.g., Ref. (208)]. Furthermore, MMN amplitude, shown to be impaired as a function of PTSD, successfully indexes auditory improvements in schizophrenia patients after cognitive training (209); additional research is warranted to determine if auditory training can enhance attentional focus in PTSD patients, and if MMN amplitude change reflects hypothesized success in training. Individuals with PTSD, especially those exposed to combat or trauma specifically linked to auditory signals (e.g., bombs or explosions) should be first referred for hearing tests to determine whether basic auditory capacity is impaired and can be remedied.

On the whole, many patients with PTSD show attentional bias to visual stimuli relevant to the trauma they have experienced. As prolonged exposure therapy shows success in reducing PTSD symptoms [e.g., Ref. (210)], P2, P3, and LPP amplitude pre-, during-, and post-treatment may effectively track habituation to trauma and/or predict at the outset who would improve the most from exposure therapy. These ERP metrics may also predict or measure success of other treatments, including attentional bias modification therapy, which specifically targets exaggerated threat monitoring and significantly reduces PTSD risk after combat trauma (211). A subset of patients devote less neural resources to the processing of task-relevant visual stimuli, independent of trauma, reflected by small target P3b amplitudes; identifying individuals with this specific deficit and referring them to cognitive training enhancing visual attention may improve goal achievement and overall life functioning.

Identifying an individual’s strengths and weaknesses with respect to attention, working memory, inhibition, and reward/emotion processing using EEG/ERPs may determine what types of treatment are warranted; particular patients may benefit from more generalized cognitive training, brain stimulation, and/or neurofeedback in addition to trauma-focused therapies such as exposure.

## Conclusions

There is promise for designing interventions to more directly target EEG biomarkers related to PTSD and/or specific symptom dimensions to enhance clinical outcomes. Future clinical neuroscience research can build upon these results by: 1) further evaluating the clinical utility of CNV, MMN, P3a, P3b, and frontal/parietal alpha asymmetry as predictive and prognostic biomarkers of PTSD symptom course and treatment outcome; and 2) targeting biological and psychological processes involving frontoparietal attention networks in individuals suffering from PTSD-related symptoms.

## Author Contributions

MB and EE reviewed articles, compiled relevant data into tables, summarized main findings of each article, and provided feedback on versions of the manuscript. RA and VN provided feedback on figures, tables, interpretation of results, and multiple versions of the manuscript. JS checked data entered into tables, created the figures, wrote the first draft of the manuscript, and formatted the manuscript for journal submission.

## Funding

This work was supported by The William K. Warren Foundation.

## Acknowledgments

Dr. Stewart wishes to thank students from the Research on Anxiety, Addiction, and Depression lab at Queens College who motivated this review.

## Conflict of Interest Statement

The authors declare that the research was conducted in the absence of any commercial or financial relationships that could be construed as a potential conflict of interest.
